# Sleep deprivation of rats increases postsurgical expression and activity of L-type calcium channel in the dorsal root ganglion and slows recovery from postsurgical pain

**DOI:** 10.1186/s40478-019-0868-2

**Published:** 2019-12-23

**Authors:** Qi Li, Zi-yu Zhu, Jian Lu, Yu-Chieh Chao, Xiao-xin Zhou, Ying Huang, Xue-mei Chen, Dian-san Su, Wei-feng Yu, Xi-yao Gu

**Affiliations:** 1 Department of Anesthesiology, Renji Hospital, School of Medicine, Shanghai Jiaotong University, Shanghai, 200127 China; 2 Department of Anesthesiology, The Second Affiliated Hospital of Jiaxing University, Jiaxing, 314000 Zhejiang China; 3 Department of Anesthesiology, First Hospital of Quanzhou, Fujian Medical University, Quanzhou, 362000 Fujian China

**Keywords:** Persistent postsurgical pain, Sleep disturbance, Dorsal root ganglion, DRG, L-type channel

## Abstract

Perioperative sleep disturbance is a risk factor for persistent pain after surgery. Clinical studies have shown that patients with insufficient sleep before and after surgery experience more intense and long-lasting postoperative pain. We hypothesize that sleep deprivation alters L-type calcium channels in the dorsal root ganglia (DRG), thus delaying the recovery from post-surgical pain. To verify this hypothesis, and to identify new predictors and therapeutic targets for persistent postoperative pain, we first established a model of postsurgical pain with perioperative sleep deprivation (SD) by administering hind paw plantar incision to sleep deprivation rats. Then we conducted behavioral tests, including tests with von Frey filaments and a laser heat test, to verify sensory pain, measured the expression of L-type calcium channels using western blotting and immunofluorescence of dorsal root ganglia (an important neural target for peripheral nociception), and examined the activity of L-type calcium channels and neuron excitability using electrophysiological measurements. We validated the findings by performing intraperitoneal injections of calcium channel blockers and microinjections of dorsal root ganglion cells with adeno-associated virus. We found that short-term sleep deprivation before and after surgery increased expression and activity of L-type calcium channels in the lumbar dorsal root ganglia, and delayed recovery from postsurgical pain. Blocking these channels reduced impact of sleep deprivation. We conclude that the increased expression and activity of L-type calcium channels is associated with the sleep deprivation-mediated prolongation of postoperative pain. L-type calcium channels are thus a potential target for management of postoperative pain.

## Introduction

Surgical patients often report pre- and post-surgical sleep disorders, and this is mainly due to anxiety, depression, stress, and the use of opioids [12]. Perioperative sleep disturbance is also a risk factor for persistent pain after surgery [51]. Wright et al. examined presurgical sleep efficiency and found that patients with poor sleep on the night before surgery reported greater pain 1 week after surgery [50]. Another study of postsurgical sleep examined 75 orthopedic patients who underwent major surgery and reported similar results. Most patients in this study (89.3%) experienced pain at the surgical site, reported a Visual Analogue Scale (VAS) pain score of “4” or “5” (range: 0 to 10), said the pain persisted at least 3 days, and declared that this pain are usually accompanied by extremely poor sleep quality [5]. These and other studies thus indicate that pre- and post-surgical sleep disturbance affects postsurgical pain.

Persistent postsurgical pain after healing of a surgical incision, which has an incidence of about 10%, is a significant clinical problem. More than 320 million people worldwide undergo surgery every year, and persistent postsurgical pain is a significant public health issue [17]. This pain can be severe enough to cause serious functional impairment or even disability resulting in decreased quality of life [33]. As the population ages and the number of surgeries continue to increase, persistent postsurgical pain will become an increasingly serious problem. Long-term pain after surgery can increase use of health resources, and thereby greater disability and suffering [26]. Therefore, there is an urgent need to understand the mechanism of persistent postsurgical pain and to find new predictors and therapeutic targets to prevent and control persistent postsurgical pain.

Previous studies have examined the mechanisms underlying the transition from acute pain to chronic pain in an effort to prevent the development of persistent postsurgical pain, but there has been little clinical progress. Previously, clinicians believed that peripheral nerve injury during surgery was the major cause of persistent postsurgical pain. However, many surgical patients have symptoms of nerve damage but report no pain. For example, after osteotomy of the mandible, only about 10% of patients with severe neurological injury (partial axonal trigeminal nerve lesion) during surgery have clinically significant neuropathic pain, and the others manifests as numbness and paresthesia. Therefore, nerve injury alone cannot explain the extended duration of acute pain after surgery [22].

The dorsal root ganglion (DRG) is the key to neurotransmission between the peripheral and central nervous systems. Previous studies confirmed that neuropeptides and ion channels, such as calcium channels, in DRG neurons control sensory responses and pain [25, 44, 52]. Recent large-scale and high-quality trials have demonstrated that gabapentin and pregabalin can reduce postsurgical pain and improve sleep quality [16]. These agents inhibit the α_2_δ subunit of voltage-gated calcium channels (VGCCs), thus suggesting that these channels play an important role in the development of persistent postsurgical pain. VGCCs are essential for the physiological activities of excitable cells, including neurons, and analysis of their biophysical properties has led to their classification as low-voltage-activated (LVA) channels and high-voltage-activated (HVA) channels. Depending on the Ca_v_α_1_ subunit, HVA channels can be classified as N-, L-, R-, or P/Q-type channels. Changes in the expression and function of these channels can affect the development and persistence of several pain states [28]. However, little is known about the role of HVA channels in the development and persistence of postsurgical pain, or about the clinical effects of changes in the expression and function of these channel proteins.

To mimic the effect of reduced sleep time in experimental animals, previous researchers have used different types of mild stimuli to keep these animals awake for a long time, such as rapid eye movement sleep deprivation (REM-SD) [11, 43] and sleep restriction (SR) [45]. There is evidence that long-term continuous or intermittent REM-SD in naive experimental animals significantly increases their hyperalgesia to heat and pressure stimulation [39]. In contrast, short-term sleep deprivation does not affect basal pain perception, but it does increase the sensitivity to postsurgical painful stimuli [48]. However, the mechanism of short-term sleep deprivation on postsurgical pain hypersensitivity is not fully understood. We therefore examined the effect of a short-term sleep deprivation on postsurgical pain by using a previously described sleep deprivation procedure [19, 37].

In the present study of male Sprague Dawley rats, we sought to understand the role of HVA channels in the delayed recovery from postsurgical pain and to find a new therapeutic target for reducing prolonged postsurgical pain. Our basic approach was to implement a perioperative SD procedure and to study its effect on postsurgical pain. We also studied the expression and function of various subtypes of HVA channels in the dorsal root ganglia (DRG) during the development of postsurgical pain.

## Materials and methods

### Animal preparation

All protocols were approved by the Animal Care and Use Committee of Renji Hospital, Shanghai Jiao Tong University School of Medicine, Shanghai, China (Chairman: Dr. Huili Dai) on 20 August 2018 (Permit Number: RJ 2018–0820). All procedures followed the guidelines of the National Institutes of Health (NIH) Guide for the Care and Use of Laboratory Animals (Department of Health and Human Services, NIH Publication No. 86–23, revised 1985) and the policies of the International Association for the Study of Pain regarding the use of laboratory animals. Efforts were made to minimize suffering due to surgery and to reduce the overall number of animals. All experiments were performed on male Sprague Dawley rats (weight: 200 to 250 g) that were housed in an animal facility, and provided with ad libitum water and standard laboratory food pellets. Rats (*n* = 162) were habituated to their environment (22 to 24 °C; 50 to 60% relative humidity; 12-h light/dark cycle) for 3 days before the experiments.

### DRG microinjection

To specifically knock-down the expression of L-type calcium channels in the L4/5 DRG, which are responsible for the transmission of nociceptive information and thus conduct pain perception in the plantar incision model, we performed direct DRG microinjection with the recombinant adeno-associated virus 2/5 (AAV2/5) with Ca_v_1.2 (Cacna1c) shRNA. DRG microinjection was performed as described previously, with minor modification [29, 56]. Briefly, a midline incision was made in the lower back of the lumbar spine to reveal L4 and/or L5 articular processes, which were then removed with a bone rongeur. After the DRG was exposed, a viral solution (1.62 × 10^13^ Vector Genomes (V.G.)/mL, 2 μL) was injected into two sites of L4 and L5 DRG using a glass micropipette connected to a Hamilton syringe. The pipette was removed 10 min after injection. The surgical field was rinsed with sterile saline and the skin incision was closed with sutures. The injected rats displayed no sign of paresis or other abnormalities, indicating that immune responses to the viral injections were minimal.

The viral solution consisted of AAV2/5-H1-shRNA (Cacna1c)-CAG-EGFP or AAV2/5-H1-NC_shRNA-CAG-EGFP-WPRE-pA (Taitool Bioscience Co.Ltd., Shanghai, China). The viral vector was pAAV2/5-H1-shRNA-CAG-EGFP [53]. H1 was the promoter for shRNA, and CAG was the promoter for EGFP. The supplemental materials provide detailed viral vector mapping and sequencing data. The sequence of Ca_v_1.2(Cacna1c) shRNA was: 5′- TCCCCgCCATTTTCACCATTGAAATTTTCAAGAGAAATTTCAATGGTGAAAATGGcTTTTT-3′. AAV2/5-H1-NC_shRNA-CAG-EGFP-WPRE-pA was used as a negative control to eliminate the influence of other interfering factors. Each recombinant AAV2/5 was locally injected into L4 and L5 DRG 21 days before SD, because AAV2/5 requires about 3 weeks before beginning gene expression after injection, and maintains relatively long-term gene transcriptional expression ability as an episome [30]. To confirm the positive and control AAVs effectively infected the DRG neurons, frozen sections of L4 and L5 microinjected DRG was observed to detect the presence of abundant green fluorescence (EGFP). A non-injected DRG was used to exclude non-specific emission and to account for background fluorescence (Additional file 1: Figure S4e). There were 5 to 10 rats per group.

### Postsurgical pain model

The plantar incision (PI) surgery was performed as previously described [4]. Rats were anesthetized with 2% isoflurane, with 0.8–1.0 L/min oxygen delivered via a nose cone. The surface of the left hind paw was prepared under sterile conditions. Then a 1-cm longitudinal incision was made with a surfical blade through the skin and fascia of the plantar aspect of the foot, starting 0.5 cm from the proximal edge of the heel and extending toward the toes. The origins and insertions of muscles remained intact, and the flexor muscle was elevated and incised. After hemostasis with gentle pressure, the skin was sutured with 5–0 nylon thread and the wound was covered with bacitracin ointment. After surgery, the animals were allowed to recover in their cages. Typically, the wounds healed well within 5 to 6 days. In all experiments, the contralateral paw was used as a control. Rats in a sham control group received anesthesia but no surgery.

### Sleep disturbance procedure

Rats were intermittently deprived of REM sleep using the small-platform method, as described previously [19, 37]. In brief, a small platform (15 cm high, 5 cm diameter) that was fixed to the center of a plastic water tank cage (45 × 53 × 72 cm) was surrounded with water (5 cm deep). At the onset of sleep, the muscular atonia caused the body to contact the water, thus awaking the animal. Each rat was placed individually on a platform within a plastic water tank cage, and was housed therein for 6 h per day for 3 consecutive days during the daytime before and after the surgery (6 days total), with food and water supplied ad libitum. Control rats were placed in groups in plastic cages in the same environment. For most experiments, rats were divided into 4 groups (5 to 10 rats per group): sham; sham+ SD; incision; and incision+SD. Some experiments employed additional treatments with nifedipine, (an L-type channel sensitive calcium channel blocker) or viral injections, as indicated in the text.

### Behavioral tests

#### von Frey filaments (mechanical stimulation)

The von Frey filament test was performed each day from 1 day before surgery to 15 days after surgery. Each rat was habituated in a small (7.5 × 15 × 15 cm) plastic cage with air vents at the top for at least 30 min before testing. Mechanical sensitivity was determined with a series of von Frey filaments (2.0 to 26 g) that were applied to the plantar surface of the left and right hind paws. Each filament was tested five times in increasing order from the lowest force. Between individual measurements, von Frey filaments were applied at least 3 s after the rats had returned to their initial resting state. The minimal force that led to either a rapid paw withdrawal and/or an escape attempt in at least 3 of the 5 stimulations was determined as the threshold of the mechanical response.

#### Laser heat pain (thermal stimulation)

Each rat was habituated for 30 min in a small plastic cage (7.5 × 15 × 15 cm) with air vents at the top on a glass plate. Laser heat was applied by aiming a beam of light through a hole in the light box, through the glass plate, to the middle of the plantar surface of each hind paw. When the animal lifted its foot, the light beam was turned off. The time from stimulation to foot withdrawal (latency) was measured. Each trial was repeated three times at 10-min intervals for each hind paw, and a cut-off time of 20 s was used to avoid tissue damage.

### Drug application

Nifedipine (N7634, Sigma), a small molecule L-type channel-sensitive calcium channel blocker which is widely used in clinical practice, was dissolved in a vehicle solution of 95% sterile saline and 5% DMSO. Then, intraperitoneal injections (15 mg/kg; a concentration that produces an antinociceptive effect in rats [49]) were given 1 h before the behavioral tests. Nifedipine works within 10 min after administration, has maximal effect in 1 to 2 h, and its effect lasts 4 to 6 h. Normal saline (NS) was injected into control rats. Each injection was given in a volume less than 1.0 mL on days 8 and 9 after incision surgery.

### Western blotting

Western blot analysis was performed as previously described, with minor modification [3, 56]. In brief, bilateral L4–6 DRG were collected, rapidly frozen, and homogenized in chilled SDS lysis buffer (P0013G, Beyotime). The crude homogenate was centrifuged at 4 °C for 15 min at 12,000 *g*. The supernatant was collected and the pellet (nuclei and debris) was discarded. Protein concentration was measured, and the samples were then heated at 100 °C for 15 min and electrophoresed in SDS-PAGE. The proteins were then transferred onto polyvinylidene fluoride (PVDF) membranes (IPVH00010, Immobilon-P). The membranes were blocked with 1% bovine serum albumin (BSA) at 4 °C overnight, and then incubated with rabbit anti-Ca_v_1.2 antibody (L-type; 1:200, ACC-003, Alomone), rabbit anti-Ca_v_2.1 antibody (P/Q-type; 1:200, ACC-001, Alomone), rabbit anti-Ca_v_2.2 antibody (N-type; 1:200, ACC-002, Alomone), rabbit anti-Ca_v_2.3 antibody (R-type; 1:200, ACC-006, Alomone), rabbit anti-Ca_v_3.2 antibody (T-type; 1:200, ACC-001, Alomone), mouse anti-Egr1 antibody(1:200, sc-101,033, Santa Cruz), or rabbit beta tubulin antibody (1:3000, AB0039, Abways) at 4 °C overnight under gentle agitation. Beta tubulin was used as a loading control. The membranes were washed and then incubated with a horseradish peroxidase-conjugated goat anti-rabbit secondary antibody (1:2000, A0208, Beyotime) or a horseradish peroxidase-conjugated goat anti-mouse secondary antibody (1:2000, A0216, Beyotime) for 1 h at room temperature. The blots were developed using the ECL Plus detection system. Band density was measured using Image J software.

### Immunofluorescence

Tissues were collected from a separate group of for immunofluorescence studies. Rats were subjected to perfusion with 4% paraformaldehyde (PFA) in phosphate-buffered saline (PBS), followed by 4% PFA in PBS post-fixation overnight. The L4–6 DRG were cryo-protected in a 20% sucrose solution overnight, and then in a 30% sucrose solution. The tissues were dissected and processed (section thickness: 20 μm) for immunofluorescence staining as previously described [14, 31]. Sections were intensively washed with PBS, and then treated with an immunostain blocking buffer (P0102, Beyotime) for 1 h at room temperature before staining. The primary antibody was rabbit anti-Ca_v_1.2 antibody (1:50, ACC-003, Alomone). Double staining used the rabbit anti-Ca_v_1.2 antibody with an antibody against FITC-conjugated isolectin B4 (marker for small non-peptidergic neurons [32]; 10 μg/mL, L2895, Sigma); an antibody against mouse anti-CGRP (marker for small peptidergic neurons [32]; 1:100, ab81887, Abcam); an antibody against mouse anti-neurofilament 200 (marker for medium/large neurons [32]; 1:500, N0142, Sigma); and mouse anti-Egr1 antibody to detect whether Egr-1 and Ca_v_1.2 are present in the same cell (1:200, sc-101,033sc-101033, Santa Cruz). All sections were then incubated with either anti-mouse IgG conjugated to Alexa Fluor® 488 (1:1000, 4408, Cell Signaling) or anti-rabbit IgG conjugated to Alexa Fluor® 594 (1:1000, 8889, Cell Signaling). Conjugated antibodies were also used for nuclear staining with DAPI. Images were taken with a fluorescence microscope (Olympus, DP80) and processed using Image J software. Three to four slices per DRG per rat were counted to eliminate the uneven distribution of large, medium and small neurons due to the irregular shape of DRG [30]. There were 3 to 4 rats per treatment group.

### Whole-cell patch clamp recording

#### DRG neuron culture

Acute dissociated L4–5 DRG neurons were prepared as previously described [30]. Rats were divided into groups as described above, and then euthanized with isoflurane. The L4–5 DRG were collected in cold DMEM/F12 medium (12634–010, Gibco) with 10% fetal bovine serum (10099-141, Gibco), 100 U/mL Penicillin, and 100 μg/mL Streptomycin (15140-122, Gibco), and then treated with an enzyme solution (5 mg/mL dispase and 1 mg/mL collagenase type I) in HBSS (14025-076, Gibco). Neurons were dissociated after trituration, resuspended in mixed DMEM/F12, and then plated onto 5 mm diameter coverslips that were coated with 50 μg/mL poly-D-lysine (P0296, Sigma). The DRG neurons were incubated at 95% O_2_ and 5% CO_2_, and at 37 °C.

#### HVA calcium channel current recording

Whole-cell patch clamp recording was performed 3 to 8 h after plating. Coverslips were placed in the perfusion chamber. The electrode resistances of the micropipettes ranged from 4 to 6 MΩ. Neurons were voltage-clamped with an Axon 1550B amplifier using Clampex software [30, 56]. The Cold Spring Harbor Protocol was followed to separate HVA calcium current from L-type calcium current [15]. The intracellular pipette solution (pH 7.3 with CsOH, 290 mOsm) contained 110 mM CsCl, 5 mM MgCl_2_, 10 mM EGTA, 10 mM HEPES, 4 mM Mg-ATP, and 0.1 mM GTP. The extracellular solution (pH 7.3 with TEA-OH, 300 mOsm) contained 5 mM CaCl_2_, 130 mM tetraethylammonium chloride (TEA-Cl), 0.3 mM TTX, 10 mM HEPES, and 10 mM glucose. Series resistance was compensated by 60 to 80%. After establishment of a giga-Ω seal, the neuron membrane potential was maintained at − 90 mV. An initial depolarizing step was applied to change the holding potential to − 30 mV for 1 s to inactivate all LVA calcium channels. Then, a second depolarizing step to 0 mV for 100 ms was applied so that only HVA calcium channels were activated [15]. Online P/4 leak subtraction was performed to eliminate this effect. All data were stored and analyzed using Clampfit software [30, 56]. To specifically verify the contribution of L-type current to the total HVA current, 1 μm nifedipine was applied to the neurons via bath perfusion during measurements.

#### Action potential recording

To record the action potential, the recording mode was switched to the current clamp. The extracellular solution (pH 7.38 by NaOH) contained 140 mM NaCl, 4 mM KCl, 2 mM CaCl_2_, 2 mM MgCl_2_, 10 mM HEPES, and 5 mM glucose. The intracellular pipette solution (pH 7.38 with KOH, 300 mOsm) contained 135 mM KCl, 3 mM Mg-ATP, 0.5 mM Na_2_ATP, 1.1 mM CaCl_2_, 2 mM EGTA, and 5 mM glucose. The resting membrane potential was recorded 3 min after a stable recording was first obtained. Depolarizing currents of 100 to 1400 pA (200-ms duration) were delivered in increments of 100 pA until an action potential (AP) occurred. The injection current threshold was defined as the minimum current needed to evoke an AP. The membrane potential was maintained at the existing resting membrane potential during the current injection. The AP threshold was defined as the first point on the rapidly rising phase of the spike at which the change in voltage exceeded 50 mV/ms, and the AP amplitude was defined as the distance from the peak to the baseline. The membrane input resistance of each cell was obtained from the slope of a steady-state I–V plot in response to a series of hyperpolarizing currents (200-ms duration) that were applied in steps of 100 pA, from 200 pA to − 2000 pA. The after-hyperpolarization amplitude was the distance from the maximum hyperpolarization to the final plateau voltage, and the AP overshoot was the distance from the AP peak to 0 mV. All experiments were performed at room temperature and all data were stored and analyzed using Clampfit software [30, 56].

#### Luciferase assay

To examine the effect of Egr-1 on the activity of the Ca_v_1.2 promoter, a luciferase assay was performed. A fragment from the *Cacna1c* gene promotor region and a fragment from the *Egr-1* gene were amplified by PCR from genomic DNA to construct *Cacna1c* gene reporter plasmids and the *Egr-1* gene over-expression plasmids, respectively. The PCR products were ligated into the GV238 vector (containing the firefly luciferase reporter gene) and the GV141 vector (containing the renilla luciferase reporter gene) using KpnI and XhoI restriction sites, respectively. DNA sequencing was performed for verification. HEK-293 T (ATCC) cells were cultured for 1 day in DMEM/F12 (12634–010, Gibco) containing 10% fetal bovine serum (10099-141, Gibco) at 37 °C in a humidified incubator with 5% CO_2_. Cells were then transferred to a 24-well plate, transfected with the *Cacna1c* gene reporter plasmids with an empty GV141 vector (control) or with *Egr-1* gene over-expression plasmids using X-tremegene HP (Roche), according to the manufacturer’s instructions. Two days after transfection, the cells were collected in a passive lysis buffer. The supernatant was used to measure luciferase activity using the Dual-Luciferase Reporter Assay System (E1910, Promega). Independent transfection experiments were repeated three times. The relative reporter activity was calculated after normalization of firefly fluorescence to renilla fluorescence.

### Statistical analysis

All results are presented as means ± standard errors of the mean (SEMs). Statistical analysis was performed using Prism 7.0 software. A two-tailed, unpaired Student’s *t*-test and one-way or two-way ANOVA were used as appropriate in multiple-comparisons tests. A *P* value below 0.05 was considered significant.

## Results

### SD prolongs recovery from postsurgical pain

**Fig. 1:**
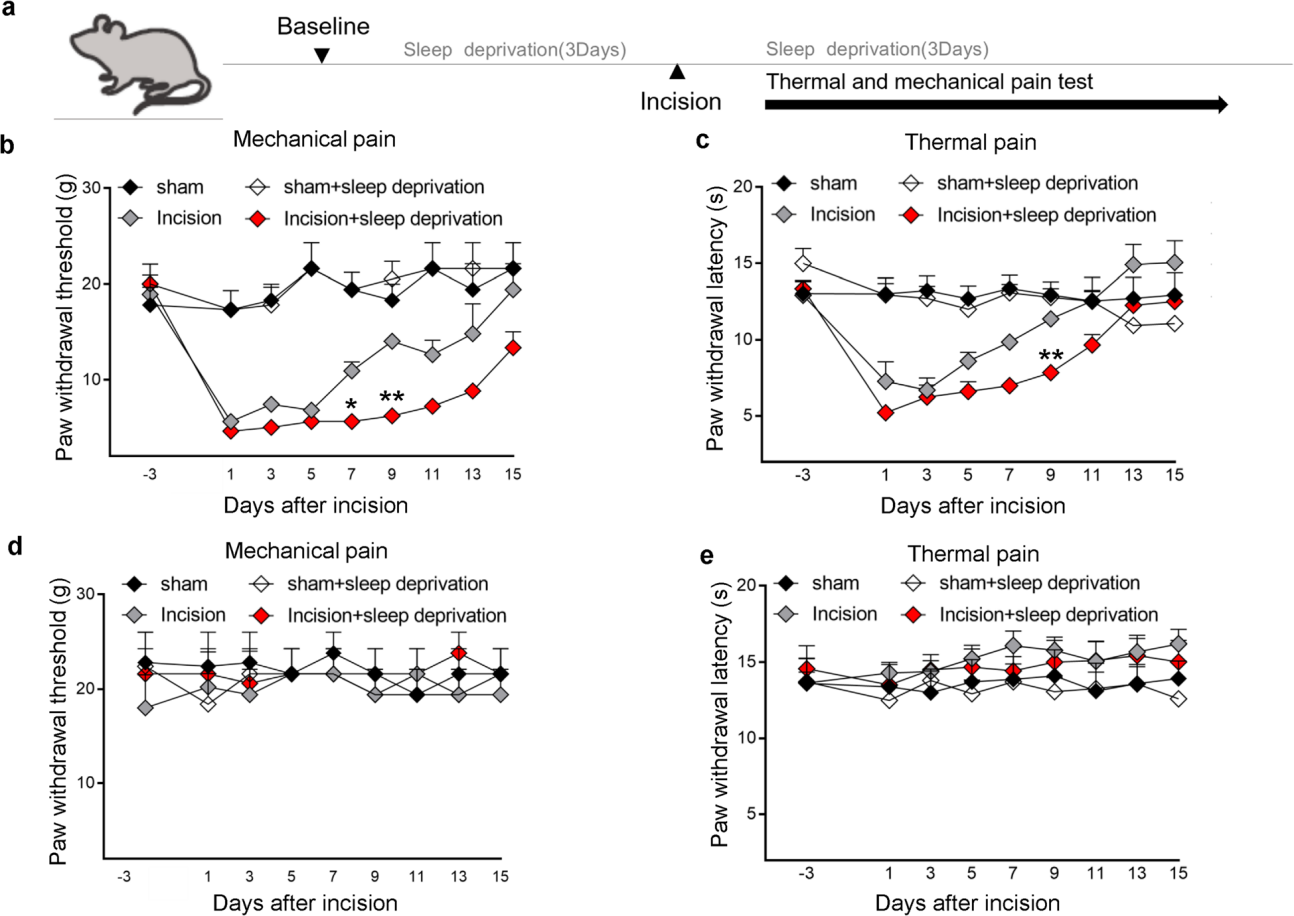
Perioperative SD prolongs postsurgical pain. **a** Design of behavior experiments. Baseline responses were measured before all interventions. SD (6 h per day) began 3 days before and ended 3 days after surgery. Paw withdrawal threshold (mechanical pain) and paw withdrawal latency (thermal pain) were measured 3 days before surgery and for 15 days after surgery. **b, c** Paw withdrawal threshold and paw withdrawal latency of rats in the sham, sham+SD, incision, and incision+SD groups (5 to 10 rats per group). Two-way ANOVA followed by a post hoc Tukey test: F (3, 242) = 82.12 for mechanical pain, F (3, 269) = 42.62 for thermal pain; *p < 0.05, **p < 0.01 for the incision group vs. the incision+SD group at each time point. **d, e** Paw withdrawal threshold and paw withdrawal latency of contralateral (control paws) in the same four groups (5 rats per group). Two-way ANOVA followed by a post hoc Tukey test: F (3, 144) = 0.9032 for mechanical pain, F (3, 144) = 4.895 for thermal pain

We first examined whether short-term SD during the perioperative period affects the recovery from postsurgical pain in rats. Thus, we applied 3 consecutive days of SD, performed unilateral hind paw plantar incision, and then applied SD again for 3 consecutive days (Fig. 1a). The incision caused continuous mechanical and thermal hypersensitivity, with dramatic declines in the threshold (mechanical pain) and latency (thermal pain) on day 1, and a gradual recovery to baseline on day 11 to day 15, depending on the treatment (Fig. 1b, c). As expected, SD alone had no effect. However, rats in the incision+SD group had a slower recovery to the baseline than those in the incision only group; at 9 days after surgery, the paw withdrawal threshold was 55.7% lower and the latency was 29.9% lower. The sham group had no significant changes to mechanical and thermal stimuli throughout the procedure (Fig. 1b, c). The contralateral (control) paws had similar responses that were close to baseline values in all four groups (Fig. 1d, e).

### SD upregulates L-type HVA calcium channels (Ca_v_1.2) in lumbar DRG neurons

**Fig. 2:**
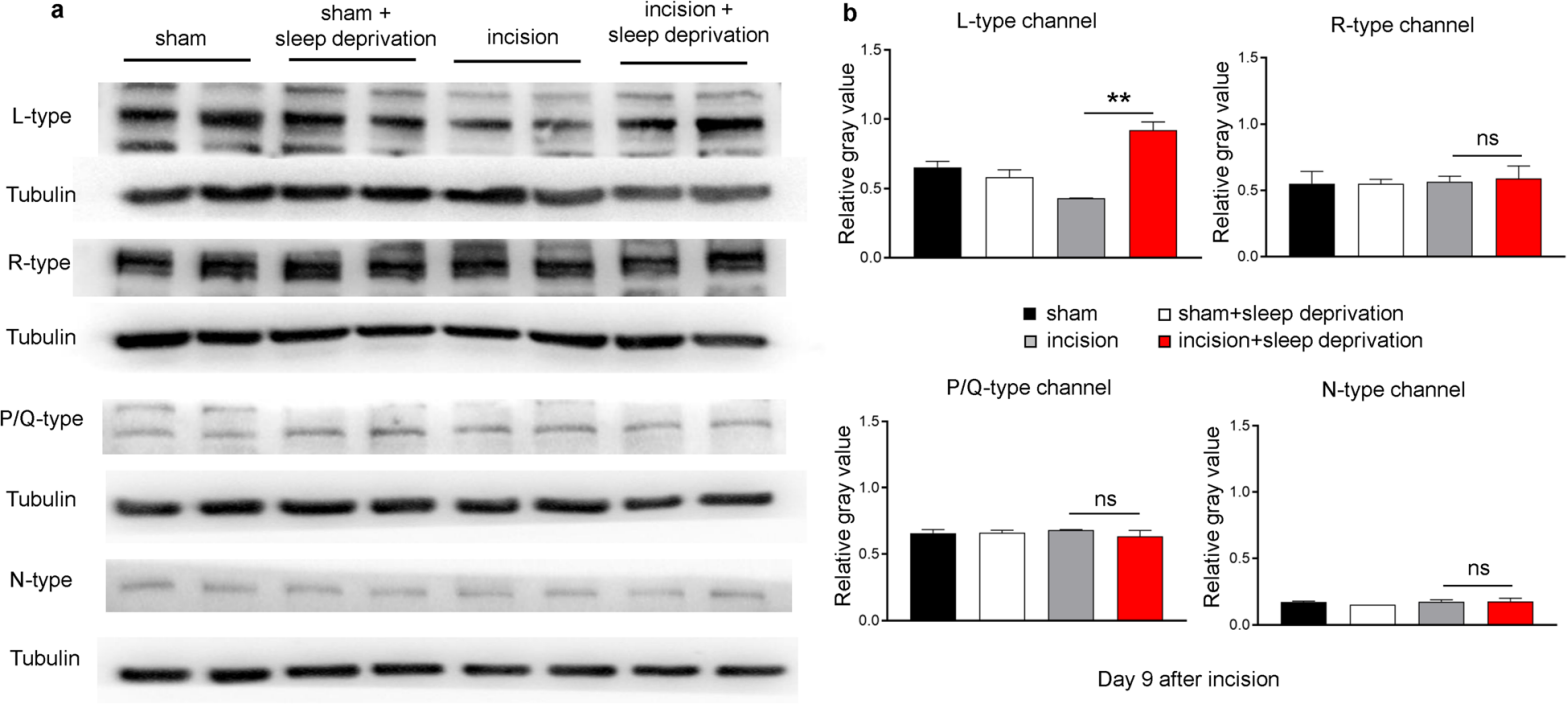
Perioperative SD increases expression of L-type HVA channels in lumbar (L4–6) DRG neurons. **a** Representative western blotting of L-type, R-type, P/Q-type, and N-type channel proteins in the L4–6 DRG of rats in the different groups on 9 days after incision. **b** Quantitation of the results in A (10 rats per group). One-way ANOVA followed by a post hoc Tukey test: F (3, 4) = 20.29 for L-type channel, F (3, 4) = 0.06732 for R-type channel, F (3, 4) = 0.4816 for P/Q-type channel, F (3, 4) = 0.6502 for N-type channel, **p < 0.01 for incision group vs. the incision+SD, ns: no significant difference)

HVA activated calcium channels in DRG neurons play critical roles in pain transmission. Thus, we hypothesized that HVA calcium channels might be altered in the DRG of rats subjected to perioperative SD. We focused on L-type channels, because they play an important role in pain, especially Ca_v_1.2, and almost 90% of the these channels are encoded by Ca_v_1.2 in nervous system [28]. At 9 days after surgery, the western blotting results indicated greater expression of L-type HVA calcium channels in the lumbar DRG of rats in the incision+SD group than in the incision only group (Fig. 2a, b). Moreover, SD alone did not change the expression of L-type channels, and there were no difference in the expression of other subtypes of HVA calcium channels (N-type, R-type, and P/Q-type) among the different groups. The four groups also had no differences in the expression of T-type channels (Additional file 1: Figure S1a, b). Our findings thus indicate that perioperative SD of rats leads to increased expression of L-type HVA calcium channels in the DRG.

### SD mainly increases expression of L-type HVA calcium channels (Ca_v_1.2) in medium and large DRG neurons

**Fig. 3:**
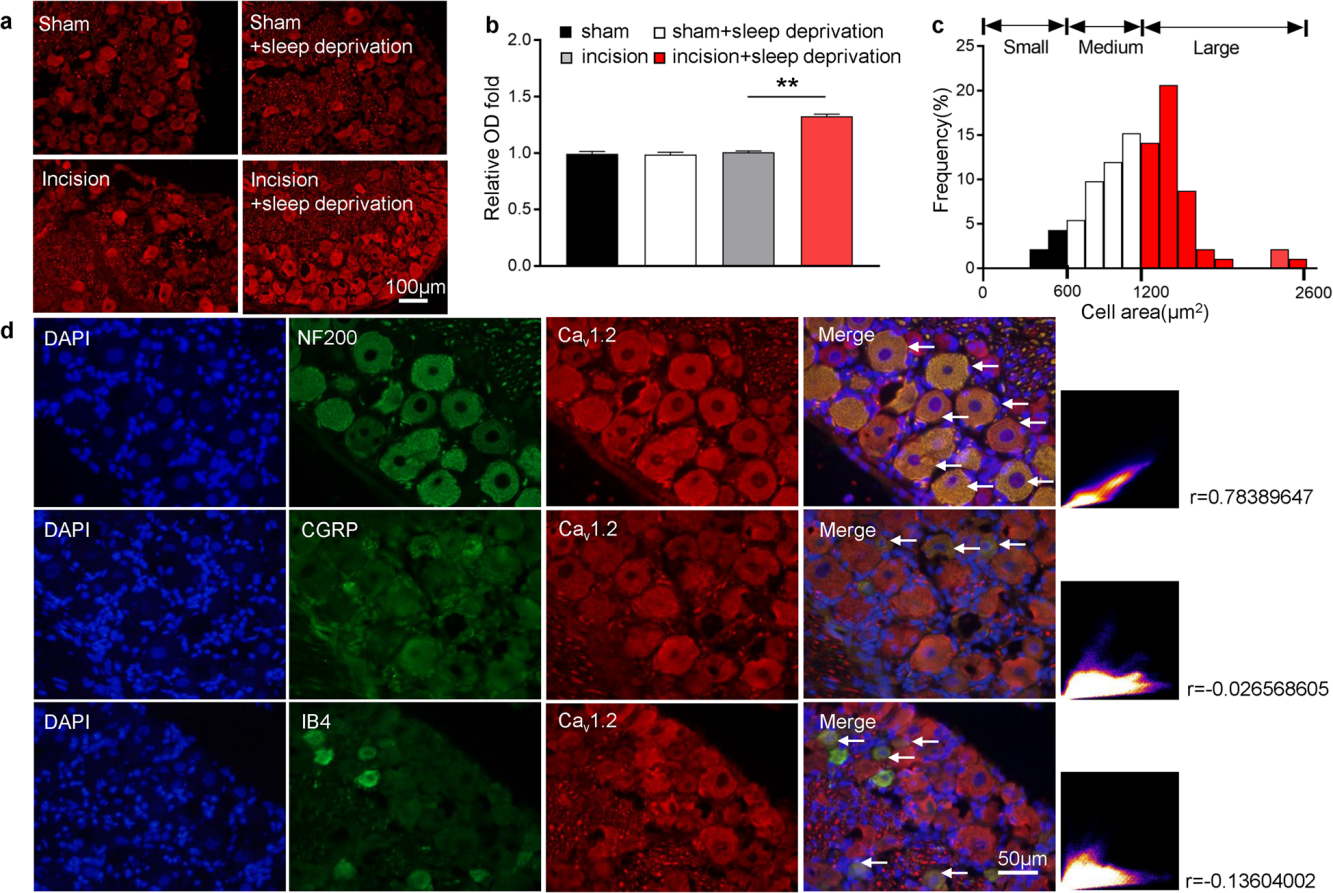
Perioperative SD increases expression of L-type HVA channels in lumbar (L4–6) DRG neurons, mainly in medium/large cells. **a** Representative immunofluorescence results, showing higher expression of L-type channels on the neuronal membranes of rats in the incision+SD group than the incision group. Scale bar: 100 μm. **b** Quantitation of the results in A (10 rats per group). One-way ANOVA followed by a post hoc Tukey test: F(3, 8) = 74.13, **p < 0.01 for the incision group vs. the incision+SD group. **c** Size distribution of L-type-channel (Ca_v_1.2)-labelled neuronal somata in the DRG (small: 6.59%, medium: 42.86%, large: 50.55%). **d** Representative immunofluorescence results showing strong co-expression of the L-type channel (Ca_v_1.2) with neurofilament 200 (top row), but little or no co-expression with calcitonin gene-related peptide (CGRP; middle row), or isolectin B4 (IB4; bottom row). Arrows indicate co-labeling. Co-labeling analysis used the colocalization finder in Image J. Pearson’s correlation coefficients (r) are indicated. Scale bar: 50 μm

We next used immunofluorescence staining to determine the cellular localization of the L-type channels in the lumbar DRG neurons. The results showed that these channels were abundant in the DRG neurons of rats in all four treatment groups, and that rats in the incision+SD group had significantly greater expression (32.8 ± 2.6%) of these channels than rats in the incision only group (Fig. 3a, b). Consistently, measurement of the cross sectional areas of neuronal somata showed that approximately 6.59% of cells with L-type channels were small (< 600 μm^2^), 42.86% were medium (600 to 1200 μm^2^), and 50.55% were large (> 1200 μm^2^; Fig. 3c).

We further examined the distribution of L-type channels by performing double immunofluorescent staining of L-type channels with a calcitonin gene-related peptide (CGRP, a marker for small peptidergic neurons), isolectin B4 (IB4, a marker for small non-peptidergic neurons), or neurofilament-200 (NF200, a marker for medium/large neurons). The results indicated that the L-type channels were mainly co-labeled with NF200 (Pearson’s r = 0.784), rather than CGRP (r = − 0.026) or IB4 (r = − 0.136; Fig. 3d). These data thus indicate that the increased level of L-type HVA calcium channels in the incision+SD group mainly occurred in medium and large DRG neurons and their axons, although L-type channels were still partially co-labeled with CGRP.

### SD increases L-type calcium current and excitability in DRG neurons

**Fig. 4:**
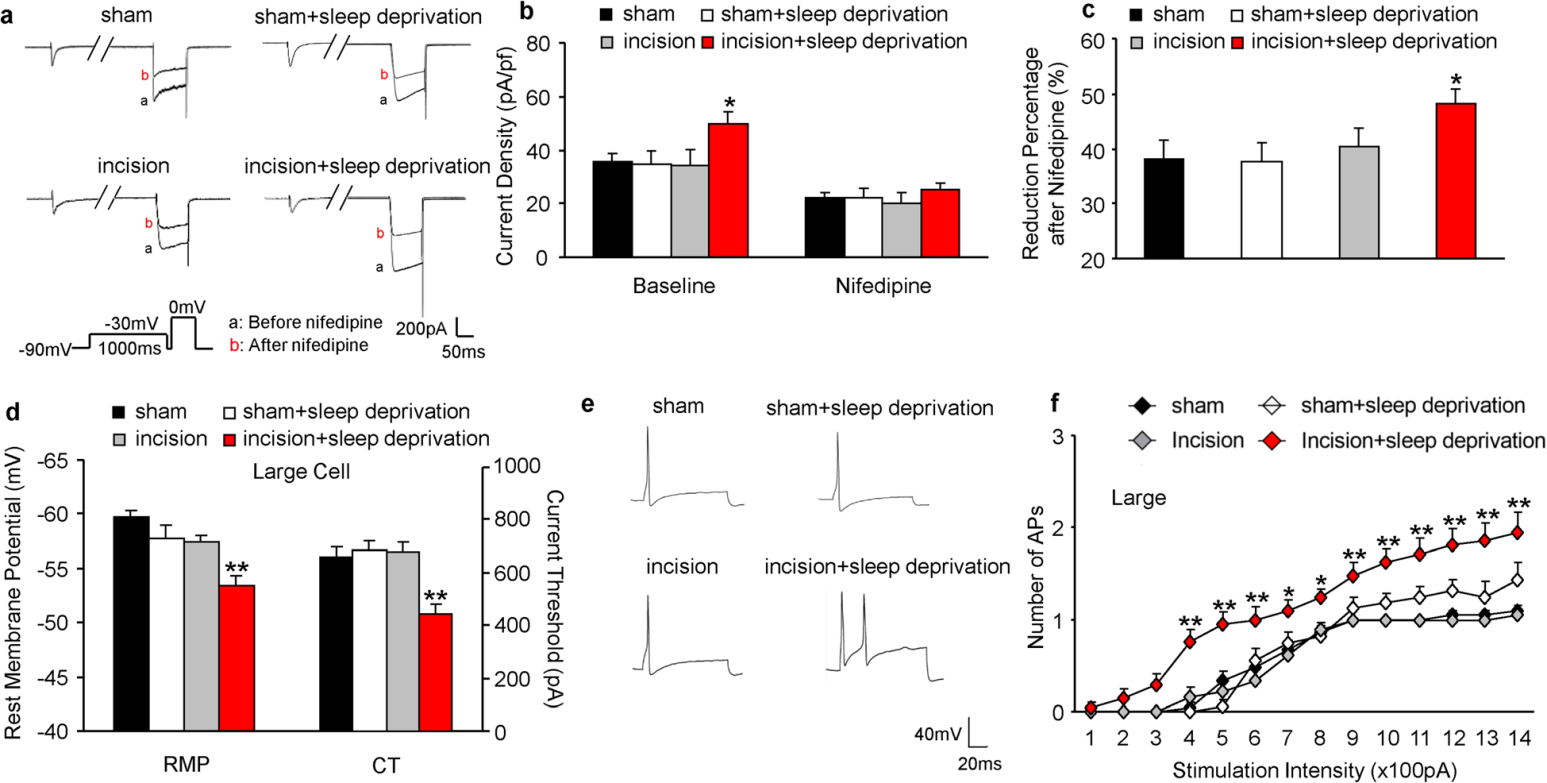
Perioperative SD increases the activity of HVA calcium channels and L-type calcium channels and excitability in large DRG neurons at 9 days after surgery. **a** Representative trace of HVA calcium channels in large DRG neurons before and after bath perfusion of 1 μm nifedipine. **b** Quantitation of the results in A (5 to 6 rats per group; 13 neurons from sham group, 15 neurons from sham+SD group, 15 neurons from incision group, 19 neurons from incision+SD group). One-way ANOVA followed by a post hoc Tukey test: F_baseline_ (3, 58) = 2.903, F_nifedipine_ (3, 58) = 0.461, *p < 0.05 for the incision group vs. the incision+SD group. **c** Effect of nifedipine on channel activity. Numbers of neurons and rats were the same as in B. One-way ANOVA followed by a post hoc Tukey test: F (3, 58) = 3.223, *p < 0.05 for the incision group vs. the incision+SD group. **d** Resting membrane potentials (left) and current threshold needed to evoke the first action potential (right) from large DRG neurons (5 to 6 rats per group, sham group: *n* = 21; sham+SD: *n* = 16; incision group: *n* = 18; incision+SD group: n = 21). One-way ANOVA followed by a post hoc Tukey test: F_RMP_ (3, 72) = 10.95; F_CT_ (3, 72) = 10.44. **p < 0.01 for the incision group vs. the incision+SD group. **e** Representative traces of evoked action potentials from DRG neurons. **f** Effect of current intensity on the number of evoked action potentials in large DRG neurons. Numbers of rats and neurons were the same as in D. Two-way ANOVA followed by a post hoc Tukey test: F_group_ (3, 1008) = 96.37, *p < 0.05, **p < 0.01 for the incision group vs. the incision+SD group at each stimulation intensity

Because L-type calcium channels are essential for neuronal excitability [28], we sought to confirm the role of L-type calcium channels in the hyperactive response to SD by measuring L-type calcium current and neuronal excitability in the DRG of rats. Whole-cell voltage-clamp recording was performed in acutely disassociated neurons from the L4/5 DRG at 8 to 9 days after surgery. The Cold Spring Harbor protocol [15] was applied to separate HVA calcium channels from L-type calcium channels. The current was stable during these recordings (Additional file 1: Figure S2a). The results indicated that HVA calcium channel current densities were significantly greater in the large, medium, and small DRG neurons of rats in the incision+SD group (Fig. 4a, b; Additional file 1: Figure S2b, d). In contrast, there were no marked differences in the HVA current from large, medium, and small neurons from the L4/5 DRG among the other 3 groups. Bath application of 1 μM nifedipine (a selective L-type calcium current inhibitor) led to greater reductions in the HVA calcium current from large, medium, and small DRG neurons in the incision+SD group than in the other 3 groups (Fig. 4c; Additional file 1: Figure S2c, e). This indicates greater activity of L-type channels in all sizes of DRG neurons of rats in the incision+SD group. DRG neurons from the sham, sham+SD, and incision groups had similar reductions in L-type channel current after nifedipine treatment (Fig. 4c; Additional file 1: Figure S2c, e).

We also performed whole-cell current-clamp recording to measure neuronal excitability from the L4/5 DRG of rats at 8 to 9 days after surgery. Compared with the incision group, large, medium, and small DRG neurons of rats in the incision+SD group had significantly less negative resting potentials (4.3, 5.3, and 5.8 mV, respectively; Fig. 4d left; Additional file 1: Figure S3a) and decreased current threshold for generation of action potential (34.6, 29.1, and 34.7%, respectively; Fig. 4d right; Additional file 1: Figure S3b). Moreover, the large, medium, and small DRG neurons of the incision+SD group had more APs than the incision group (Fig. 4e, f; Additional file 1: Figure S3c, d). However, there were no marked changes in membrane input resistance and other action potential parameters, including threshold, amplitude, overshoot, or afterhyperpolarization amplitude among the groups (Additional file 1: Table S1). These data indicate that the increased expression of L-type calcium channels in the incision+SD group was associated with increased neuronal hyperexcitability in the DRG in this group.

### Blocking L-type HVA channels in the lumbar DRG accelerates recovery from postsurgical pain in rats subjected to SD

**Fig. 5:**
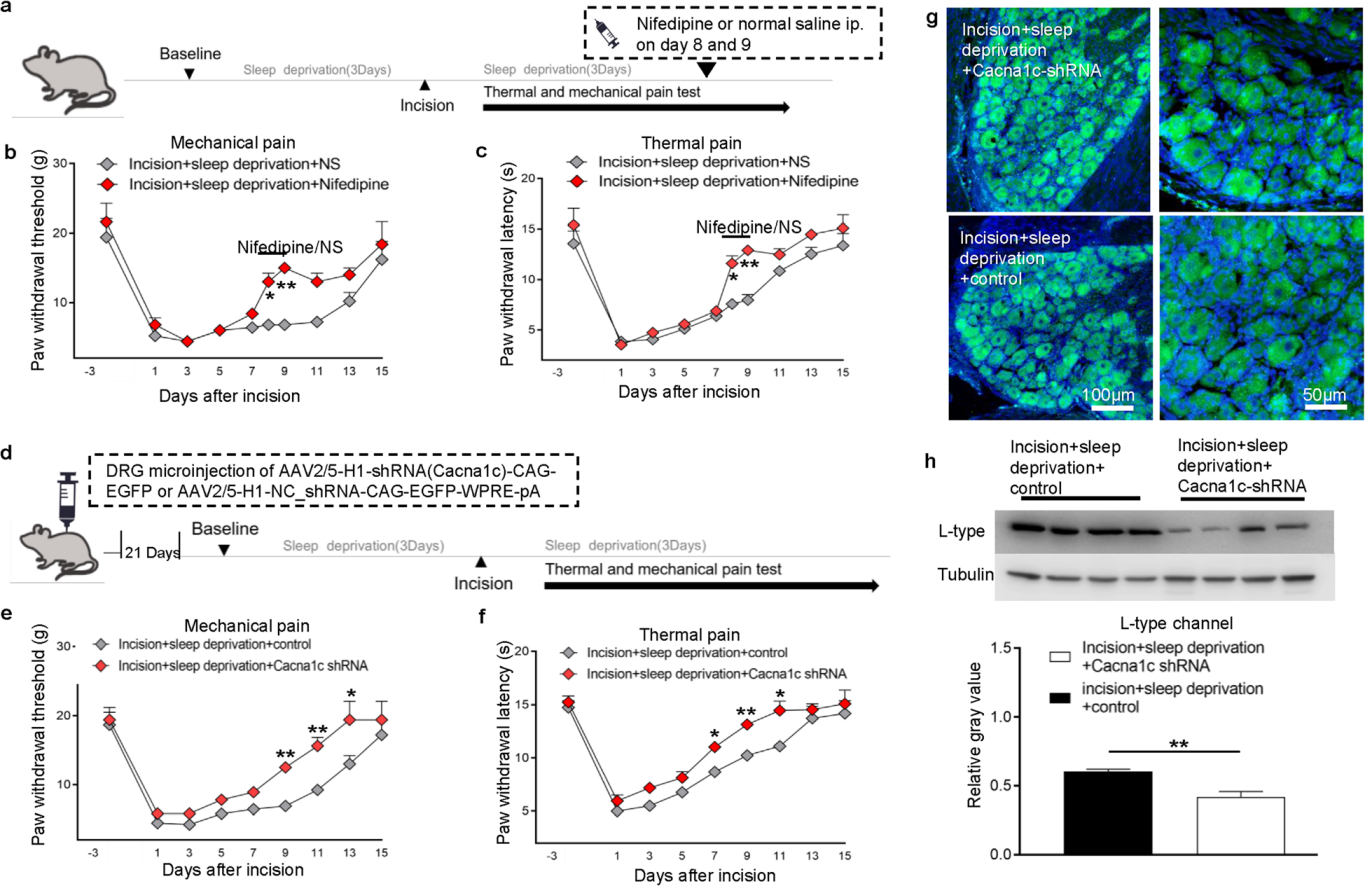
Blocking L-type HVA channels in the lumbar DRG accelerates recovery from postsurgical pain in rats subjected to perioperative SD. **a** Behavior experiments were performed as described in Fig. 1, but with intraperitoneal injection of nifedipine or solvent (normal saline, NS) on 8 or 9 days after surgery. **b, c** Paw withdrawal threshold and paw withdrawal latency were determined as described in Fig. 1 (10 rats per group) Two-way ANOVA followed by post Sidak’s multiple comparisons test: F (1, 180) = 25.47 for mechanical pain (B); F (1, 180) = 27.33 for thermal pain (C), *p < 0.05, **p < 0.01 for comparison of the incision+SD + nifedipine group vs. the incision+SD + NS group at each indicated time. **d** Behavior experiments were performed as described in Fig. 1, but with DRG injection of AAV2/5-H1-shRNA(Cacnalc)-CAG-EGFP or AAV2/5-H1-NC_shRNA-CAG-EGFP-WPRE-pA at 21 days before behavior testing, and measurement of virus expression 3 days before paw surgery. **e, f** Paw withdrawal threshold and paw withdrawal latency were determined as described in Fig. 1 (5 to 10 rats per group). Two-way ANOVA followed by post Sidak’s multiple comparisons test: F(1, 135) = 36.13 for mechanical pain (e), F (1, 126) = 38.64 for thermal pain (f), *p < 0.05, **p < 0.01 for comparison of the incision+SD + Cacna1c shRNA group vs. the incision+SD + control group at each indicated time. **g** Representative lumbar DRG immunofluorescence sections of rats with DRG infection by AAV2/5-H1-shRNA(Cacna1c)-CAG-EGFP or AAV2/5-H1-NC_shRNA-CAG-EGFP-WPRE-pA. Green fluorescence indicates expression of EGFP. **h** Expression of L-type channel protein in the L4–5 DRG of rats at 21 days after DRG injection of AAV2/5 viruses on day 9 after surgery (4 rats per group). Two tailed unpaired *t* -test: *t* = 4.388, **p < 0.01 for comparison of the two groups

To examine if the increased activity of L-type channels in the lumbar DRG is related to SD-induced postsurgical pain, we examined the effect of nifedipine-mediated blocking of these channels on the duration of postsurgical pain. Nifedipine has a well-established inhibitory effect on L-type channels and an antinociceptive effect on rats [49]. Thus, we administered intraperitoneal nifedipine or solvent into incision+SD rats on days 8 and 9 after surgery (Fig. 5a); this is the time when there was the greatest difference between the incision only and incision+SD groups (Fig. 1). As expected, incision+SD led to mechanical allodynia and thermal hyperalgesia in both groups prior to day 9. However, nifedipine administration significantly reduced both types of postsurgical pain (Fig. 5b, c). In particular, nifedipine led to a paw withdrawal threshold that was about two-fold greater (Fig. 5b) and a 59.3% increase in paw withdrawal latency (Fig. 5c). Experiments with the contralateral (control) paws led to similar results in both groups (Additional file 1: Figure S4a, b).

We then specifically blocked the L-type channels of neurons in the DRG by performing in vivo microinjections of AAV2/5-H1-shRNA(Cacna1c)-CAG-EGFP into the L4 and L5 DRG to knockdown L-type calcium channels; AAV2/5-H1-NC_shRNA-CAG-EGFP-WPRE-pA was used as a negative control. We subjected rats to SD and incision surgery 3 weeks after viral microinjection to ensure efficient virus expression (Fig. 5d). Behavioral tests indicated that rats which received AAV2/5-H1-shRNA(Cacna1c)-CAG-EGFP had reduced hypersensitivity and significantly accelerated recovery from surgery relative to the controls (Fig. 5e, f). Frozen sections of L4 and L5 microinjected DRG showed that these neurons had abundant green fluorescence, indicating that the positive and control AAVs effectively infected the DRG neurons (Fig. 5g). Furthermore, the expression of L-type calcium channels was partially but significantly decreased in rats injected with AAV2/5-H1-shRNA(Cacna1c)-CAG-EGFP, indicating that the virus specifically knocked down the expression of these channels (Fig. 5h). Experiments with the control paws led to similar results in both groups (Additional file 1: Figure S4c, d). These results indicate that L-type calcium channels in the lumbar DRG may be required for the presence and prolongation of postsurgical pain in rats subjected to perioperative SD.

### Blocking L-type HVA channels in the lumbar DRG reduces L-type calcium current and excitability of DRG neurons in rats subjected to SD

**Fig. 6:**
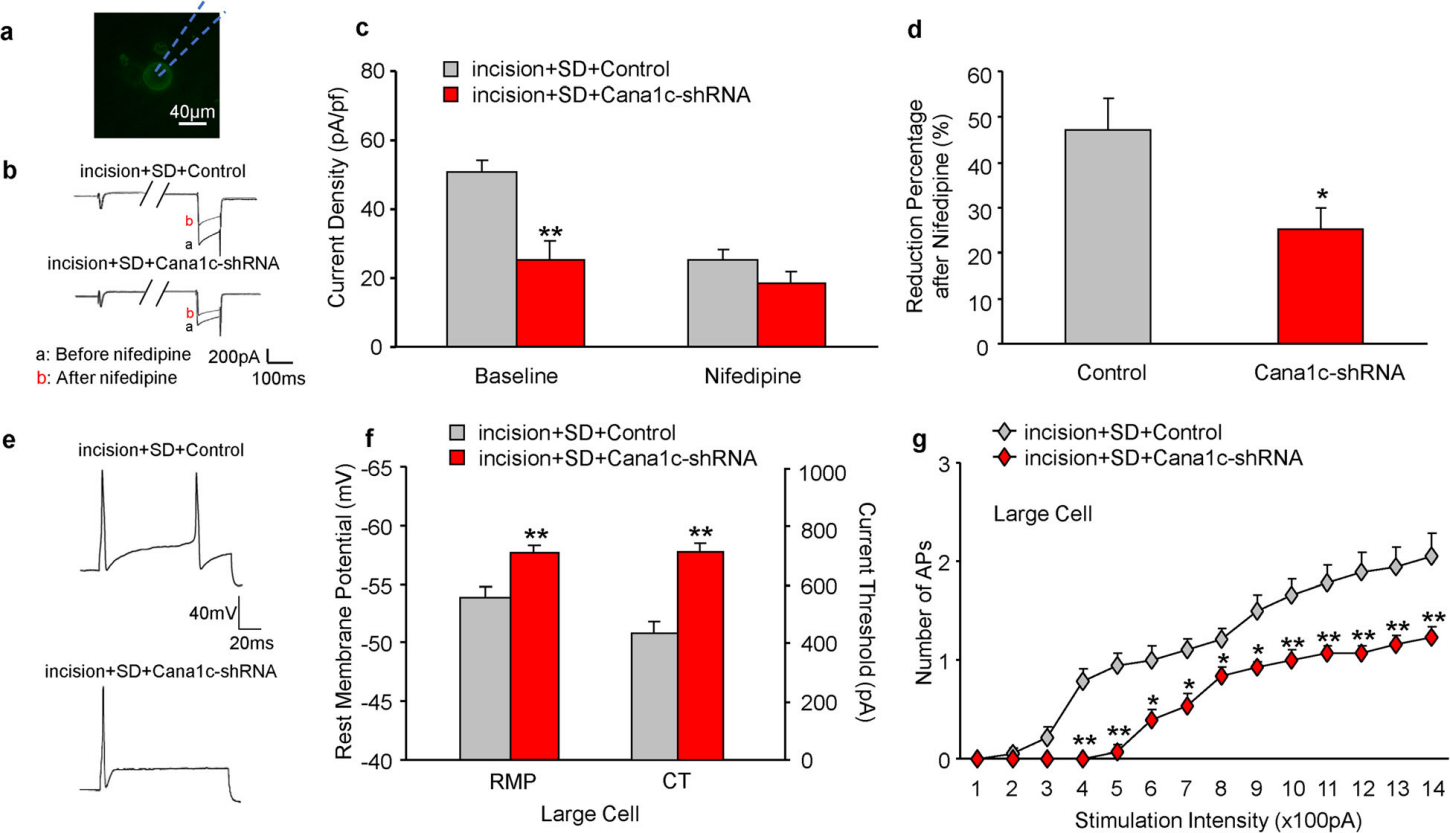
Specific knockdown of L-type HVA channels in the lumbar DRG decreases the level and activity of HVA calcium channels and hyperexcitability of large DRG neurons on day 9 after surgery in rats subjected to perioperative SD. **a** Neurons labelled with EGFP indicated successful virus transfection. **b** Representative trace of total HVA calcium channels before and after bath perfusion of 1 μm nifedipine in large DRG neurons. **c** Current density of total HVA calcium channels before and after bath perfusion of 1 μm nifedipine (5–6 rats per group; 17 neurons from incision+SD + control group; 15 neurons from incision+SD + shRNA group). Two-tailed unpaired Student’s *t*-test: *t* = 3.169, **p < 0.01 for comparison of the two groups. **d** Effect of nifedipine on percentage change in current density. Numbers of neurons and rats were the same as in C. Unpaired Student’s *t*-test: *t* = 2.497, *p < 0.05 for comparison of the two groups. **e** Representative traces of evoked action potentials from DRG neurons. **f** Resting membrane potentials (RMP, left) and current threshold (CT, right) of the first action potential generation in large DRG neurons (5 to 6 rats per group; 18 neurons from incision+SD + control group; 13 neurons from incision+SD + shRNA group). Two-tailed unpaired Student’s *t*-test: *t*_RMP_ = 2.959, *t*_CT_ = 5.283, **p < 0.01 for comparison of the two groups. **g** Effect of stimulation intensity on the number of evoked action potentials. Numbers of neurons and rats were the same as in F. Two-way ANOVA followed by a post hoc Tukey test: F_group_ (1, 406) = 118.6. *p < 0.05, **p < 0.01 for comparison of the two groups at each stimulation intensity

We further investigated whether specific blockage of the L-type calcium channels could affect the recovery of hyperexcitability of DRG neurons of rats that received incision+SD. Thus, we recorded EGFP-positive neurons at day 9 after incision (Fig. 6a), with transfection with control or shRNA virus as described above. The results indicated the AAV2/5-Cana1c-shRNA led to a significant reduction of the HVA calcium current density in large, medium, and small DRG neurons (18.6, 14.5, and 9.9 pA/pf, respectively; Fig. 6b, c; Additional file 1: Figure S5a, c). Bath application of 1 μM nifedipine further reduced the current in large, medium, and small DRG neurons of this group (Fig. 6d; Additional file 1: Figure S5b, d), thus indicating that virus microinjection led to knockdown of L-type calcium channels.

Current clamp recording also indicated that shRNA virus microinjection decreased the resting membrane potential in large, medium, and small DRG neurons (4.7, 4.3, and 4.4 mV, respectively; Fig. 6f left, Additional file 1: Figure S5e) and increased the current threshold for AP generation in all sizes of DRG neurons (276.5, 121.7, and 93.68 pA, respectively; Fig. 6f right; Additional file 1: Figure S5f). Moreover, shRNA virus microinjection in large, medium, and small DRG neurons led to a decreased number of APs in large, medium, and small DRG neurons (Fig. 6e, g; Additional file 1: Figure S5 g, h). Neither group had significant changes in membrane input resistance and other action potential parameters, including threshold, amplitude, overshoot, or afterhyperpolarization amplitude (Additional file 1: Table S2). These data indicate that specific knockdown of L-type calcium channels accelerates the post-surgical recovery of DRG neurons in rats subjected to incision+SD. L-type calcium channels thus appear to play a major role in the recovery from the perioperative SD.

### Egr-1 may trigger Ca_v_1.2 gene transcription in lumbar DRG of perioperative SD-exposed rats

**Fig. 7:**
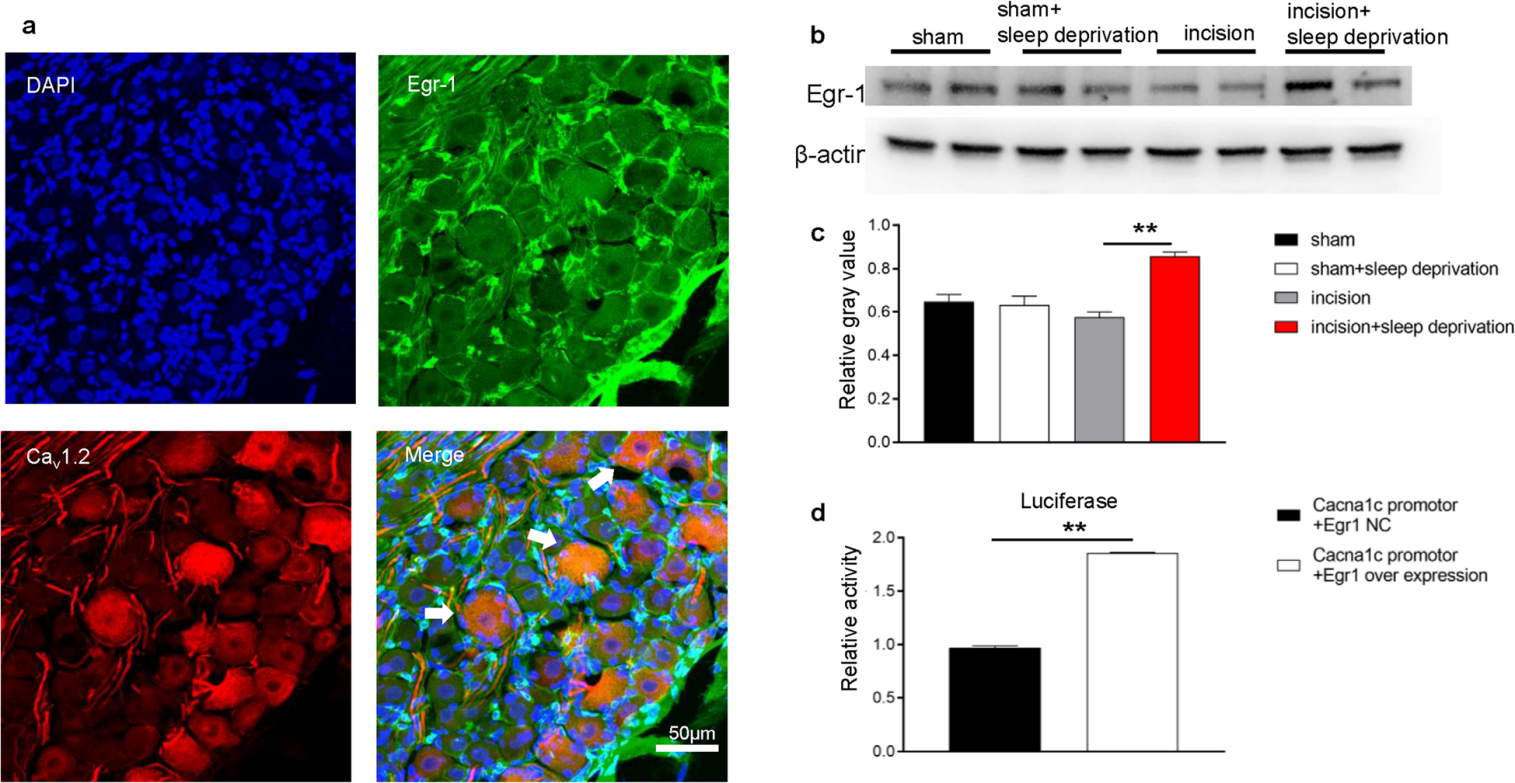
Reporter experiments indicate that Egr-1 may trigger *Cacna1c* transcription in lumbar DRG of rats that received perioperative SD. **a** Representative fluorescence images showing that L-type channel (Cacna1c) is co-expressed mostly with Egr-1. **b** Western blotting of L-type channel in the L4–6 DRG of rats in the four groups at 9 days after surgery. **c** Quantitation of the results of B (10 rats per group). One-way ANOVA followed by a post hoc Tukey test: F (3, 4) = 17.13, **p < 0.01 for comparison of two groups. **d** *Cacna1c* gene promotor activity in HEK-293 T cells transfected with the empty GV141 vectors or the Egr-1 overexpression plasmids (3 replicates per treatment). Two tailed unpaired *t* test: *t* = 54.06, **p < 0.01 for comparison of the two groups

Finally, we examined the cause of L-type channel upregulation in the lumbar DRG of perioperative SD-exposed rats. A query of the AnimalTFDB database indicated that Egr-1 might be a transcription factor of Ca_v_1.2. Thus, we speculate that Egr-1 may have such a role, because it regulates the activity of L-type channels and is also related to sleep [13, 18, 40, 46]. Our double immunofluorescence staining of L-type channels with Egr-1 showed that Egr-1 and L-type channels were mostly in the same neurons (Fig. 7a). Furthermore, the expression of Egr-1 correlated with changes in L-type channels (Fig. 7b, c), and an in vitro luciferase assay confirmed that Egr-1 overexpression significantly increased the activity of the *Cacna1c(Ca*_*v*_*1.2)* gene promoter (Fig. 7d). These data suggest that Egr-1 regulates L-type channels and plays a role in the SD-mediated delay of recovery from postoperative pain. Future studies are needed to support or refute these inferences.

## Discussion

This study of male Sprague Dawley rats demonstrated that short-term SD before and after surgery delayed recovery from postsurgical pain. In addition, our electrophysiological and molecular biology experiments indicated that prolonged postsurgical pain duration was related to the increased expression and activity of L-type calcium channels in the lumbar DRG, and that blocking these channels accelerated postsurgical recovery from pain. These results suggest that the prolonged duration of postsurgical pain that is mediated by perioperative SD depends on the expression and activity of L-type calcium channels.

L-type channels, one of the four subtypes of HVA channels, are encoded by the *Ca*_*v*_*1.1–1.4* genes. Mammals have almost no expression of Ca_v_1.1 and Ca_v_1.4 in their nervous systems, but Ca_v_1.2 and Ca_v_1.3 are expressed in most excitable cells, including neurons [28]. These proteins are present in the cell body and also in axons [9, 36], consistent with our staining results. Also, mammals with *Ca*_*v*_*1.3* knockout have a normal pain phenotype [10]. These previous results led us to focus on the L-type channels encoded by *Ca*_*v*_*1.2*. As L-type channels are widely distributed in cells that participate in the pain pathway, previous studies have also examined the role of these channels [1, 38, 41]. Increased L-type channels in spinal cord lamina II mediate hyperalgesia in rats in a chronic constriction injury (CCI) model [1]. Lack expression of L-type channels in the anterior cingulate cortex of mice correlates with pain relief [23]. The L-type channel mRNA in the cerebral cortex and thalamus is up-regulated in the presence of a migraine aura [8]. Moreover, administration of the L-type channel blocker nifedipine can enhance anti-nociceptive effect of opioids, which were microinjected into the midbrain ventrolateral periaqueductal gray (vPAG). This suggests that the activity of the L-type channels of PAG may play an important role in the development of pain [21]. In addition, L-type channels are also associated with psychological factors, such as stress, anxiety, and depression [27]. These previous studies thus indicate that L-type channels have major roles in pain occurrence and development.

Our current work demonstrated that L-type channels in the DRG contributed to the SD-induced prolongation of postsurgical pain. Our western blotting and electrophysiology data indicated increased expression and activity of L-type channels of rats that received incision+SD, and our immunofluorescent staining indicated that increased L-type channels were mainly located in medium and large neurons, but they had a lower level in small neurons. Our electrophysiological data also confirm these results. It should be noticed that although the altered L-type channels mostly located in medium and large neurons, which mainly contributed to the mechanical pain, the CGRP+ small neurons still have a proportion of co-labeling with L-type channels. According to previous reports, CGRP+ neurons mainly control thermal pain [6, 35, 54]. This is consistent with our behavioral data that the thermal pain could be partially reversed. Moreover, to rule out the effects of L-type channels at other anatomical sites in the pain pathway, we performed DRG microinjections with a recombinant AAV to specifically knockdown the L-type channels to confirm our interpretation. These findings extend the limited literature regarding the effect and mechanism of SD before or/and after surgery on recovery from postsurgical pain.

With the continuous improvement of people’s living standards, the quality of sleep has become a topic of increasing concern. Especially for surgical patients, sleep disorders often occur before and after surgery. Many studies have shown that sleep disorders and pain are closely related. In particular, sleep disorders can cause many changes in endogenous regulatory factors, which in turn can cause hyperalgesia. For example, insufficient sleep leads to increased migration of B cells into the brain compartment [24], activation of complement [47], and increased levels of IL-1 [55], thus leading to the onset and aggravation of neuroinflammation, a key factors underlying pain. In addition, stress caused by sleep disorders can cause dysfunction of the HPA axis, causing cortisol dysfunction to trigger, exacerbate, or prolong pain, impair healing, and contribute to chronic disability [20]. However, a common problem with these regulators is that specific regulation is difficult; for example, it is difficult to inhibit inflammation and regulate plasma cortisol levels to achieve pain relief. Moreover, most previous studies have focused on the relationship between sleep and pain, and few studies have examined postsurgical pain.

Our current study focused on the L-type calcium channel, and found the endogenous factor Egr-1 might have an important in the regulation of these channels in DRG. This suggests that specific regulation of the activity of Egr-1 and L-type channels has potential for the therapeutic management of peripheral postsurgical pain. Most previous studies in this field have examined the effects of SD on pain in naive animals. These studies demonstrated that long-term consecutive or intermittent SD caused abnormal nociceptive sensitivity at the basal level [11, 19]. However, it is still unclear whether short-term SD before and after surgery actually affects postsurgical pain. We established an animal model of perioperative SD, in which rats with SD had slightly greater pain sensitivity than control rats (no SD) from 1 to 5 days after surgery, although these differences were not statistically significant. This may be due to factors such as the strain of the rat, feeding conditions, and other details of the experimental model. This model thus simulates a common clinical situation, because most surgical patients have short-term SD before and after surgery, and this short-term SD does not cause changes in pain perception.

The relationship between central nervous system activity and delayed postoperative pain recovery is currently unclear. It is possible, although uncertain, whether pre- and post-surgical SD initially affects the central nervous system (CNS) and then the peripheral nervous system (PNS), eventually leading to a delay of postsurgical recovery. There is much evidence that SD causes a series of changes in the CNS. For example, mice subjected to sleep disturbance produce more Ly-6C^high^ monocytes and less hypocretin (a neuropeptide that promotes wakefulness) in the lateral hypothalamus [34]. More importantly, long-term lack of sleep in rats can lead to increased neural activity in the periaqueductal grey (PAG) and the nucleus accumbens (NAc), which are closely related to perception of pain [42]. Studies of animal models of chronic pain have identified some molecular mechanisms and neurobiological activities that are associated with the transition from acute pain to chronic pain. The most studied descending pain pathway projects from the midbrain periaqueductal grey (PAG) to the rostral ventromedial medulla (RVM). Electrical stimulation of the PAG can block the spinal cord’s response to noxious stimuli, and stimulation of the RVM can inhibit and/or promote pain signaling [57]. In addition, insufficient sleep can cause release of glucocorticoids from the adrenal gland [2] while endogenous glucocorticoids can interact with Egr-1 [7]. Therefore, we speculate that changes in glucocorticoid levels after short-term SD may also be associated with persistent postsurgical pain. Overall, we believe that a regulatory mechanism first affects the CNS and then the PNS, so that short-term SD before and after surgery delays postsurgical recovery from pain. This regulatory mechanism, which may be related to neuroimmunity, neural circuits, and/or endocrine systems, is a topic of our future studies.

A limitation of the present study is that we did not examine the effect of overexpression of L-type channels in DRG to verify that recovery from postsurgical pain is delayed upregulation and activation of these channels. To the best of our knowledge, upregulation of L-type channels leads to hyperalgesia. For example, in one animal model of chronic pain, the chronic constriction injury (CCI) model, up-regulation of L-type channels markedly decreases the pain threshold in rats. Moreover, use of L-type calcium channel blockers reduces the frequency of spontaneous excitatory postsynaptic currents, thereby providing relief from pain [1]. Thus, if we overexpressed the L-type channels in the DRG, the rats would likely remain in a constant state of hyperalgesia, and this could be difficult to distinguish from postsurgical pain. Another limitation is that we did not perform genetic knock-out of *Ca*_*v*_*1.2* to confirm our results. This was because of the technical difficulties in performing knock-out of the *Cacna1c* gene in rats. Instead, we performed lumbar DRG microinjection of Cacna1c-shRNA to specifically eliminate Ca_v_1.2 in L4/5 DRG; an advantage of our approach is that it was specific to the lumbar region. The results of our shRNA microinjection experiments confirmed that L-type calcium channels function in the prolongation of postsurgical pain.

## Conclusions

Our study confirmed that short-term sleep deprivation before and after surgery prolonged the postsurgical recovery from pain in rats, and that this response is related to increased expression and activity of L-type channels in the lumbar DRG. Partially specific blockage of the L-type channels in the lumbar DRG accelerates the postsurgical recovery from pain. Our findings may suggest that changes in the L-type channels may be related to Egr-1. Overall, our findings may help to explain why acute postsurgical pain leads to persistent postsurgical pain, and how to predict and prevent this development. Use of an alternative method of post-surgical pain management might help to reduce the social burden of the opioid crisis and improve patient quality of life after surgery.

## Supplementary information

**Additional file 1.** **Figure S1.** Expression of T-type LVA channel protein did not change in the lumbar(L4-6) DRG neurons of rats subjected to perioperative SD. **Figure S2.** Perioperative SD increases the acitivity of HVA calcium channels and L-type calcium channels in medium/small DRG neurons at 9 days after surgery. **Figure S3.** Perioperative SD increases the hyperexcitability of medium/small DRG neurons at 9 days after surgery. **Figure S4.** Blocking L-type HVA channels in the lumbar DRG with nifedipine or a spcific shRNA does not affect response in the contralateral(control) paws. **Figure S5.** Specific knockdown of L-type HVA channels in the lumbar DRG inhibits total HVA-activated calcium channels and L-type calcium channels in medium/small DRG neurons at 9 days after surgery in rats subjected to perioperative SD. **Table S1.** Membrane input resistance and other action potential parameters in DRG neurons day 9 after incision or sham surgery. **Table S2.** Membrane input resistance and other action potential parameters in DRG neurons with virus injection day 9 after incision+sleep deprivation. **Method S1.** Viral vector mapping and sequencing report.

## Data Availability

The data produced and/or analysed during the current study are available from the corresponding author on reasonable request.

## References

[1] Alles SR, Garcia E, Balasubramanyan S, Jones K, Tyson JR, Joy T, Snutch TP, Smith PA (2018). Peripheral nerve injury increases contribution of L-type calcium channels to synaptic transmission in spinal lamina II: role of alpha2delta-1 subunits. Mol Pain.

[2] Ashley NT, Sams DW, Brown AC, Dumaine JE (2016). Novel environment influences the effect of paradoxical sleep deprivation upon brain and peripheral cytokine gene expression. Neurosci Lett.

[3] Atianjoh FE, Yaster M, Zhao X, Takamiya K, Xia J, Gauda EB, Huganir RL, Tao YX (2010). Spinal cord protein interacting with C kinase 1 is required for the maintenance of complete Freund's adjuvant-induced inflammatory pain but not for incision-induced post-operative pain. Pain.

[4] Brennan TJ, Vandermeulen EP, Gebhart GF (1996). Characterization of a rat model of incisional pain. Pain.

[5] Buyukyilmaz FE, Sendir M, Acaroglu R (2011). Evaluation of night-time pain characteristics and quality of sleep in postoperative Turkish orthopedic patients. Clin Nurs Res.

[6] Chang AY, Mann TS, McFawn PK, Han L, Dong X, Henry PJ (2016). Investigating the role of MRGPRC11 and capsaicin-sensitive afferent nerves in the anti-influenza effects exerted by SLIGRL-amide in murine airways. Respir Res.

[7] Chen H, Amazit L, Lombes M, Le Menuet D (2019). Crosstalk between glucocorticoid receptor and early-growth response protein 1 accounts for repression of brain-derived Neurotrophic factor transcript 4 expression. Neuroscience.

[8] Choudhuri R, Cui L, Yong C, Bowyer S, Klein RM, Welch KM, Berman NE (2002). Cortical spreading depression and gene regulation: relevance to migraine. Ann Neurol.

[9] Clark K, Sword BA, Dupree JL (2017). Oxidative stress induces disruption of the axon initial segment. ASN Neuro.

[10] Clark NC, Nagano N, Kuenzi FM, Jarolimek W, Huber I, Walter D, Wietzorrek G, Boyce S, Kullmann DM, Striessnig J, Seabrook GR (2003). Neurological phenotype and synaptic function in mice lacking the CaV1.3 alpha subunit of neuronal L-type voltage-dependent Ca2+ channels. Neuroscience.

[11] Damasceno F, Skinner GO, Gomes A, Araujo PC, de Almeida OM (2009). Systemic amitriptyline administration does not prevent the increased thermal response induced by paradoxical sleep deprivation. Pharmacol Biochem Behav.

[12] Dimsdale JE, Norman D, DeJardin D, Wallace MS (2007). The effect of opioids on sleep architecture. J Clin Sleep Med.

[13] Donley MP, Rosen JB (2017). Novelty and fear conditioning induced gene expression in high and low states of anxiety. Learn Mem.

[14] Duan B, Cheng L, Bourane S, Britz O, Padilla C, Garcia-Campmany L, Krashes M, Knowlton W, Velasquez T, Ren X, Ross S, Lowell BB, Wang Y, Goulding M, Ma Q (2014). Identification of spinal circuits transmitting and gating mechanical pain. Cell.

[15] Gandini MA, Sandoval A, Felix R (2014). Whole-cell patch-clamp recordings of Ca2+ currents from isolated neonatal mouse dorsal root ganglion (DRG) neurons. Cold Spring Harb Protoc.

[16] Gilron I (2007). Gabapentin and pregabalin for chronic neuropathic and early postsurgical pain: current evidence and future directions. Curr Opin Anaesthesiol.

[17] Glare P, Aubrey KR, Myles PS (2019). Transition from acute to chronic pain after surgery. Lancet.

[18] Gonzalez-Ramirez R, Martinez-Hernandez E, Sandoval A, Gomez-Mora K, Felix R (2018). Regulation of the voltage-gated Ca(2+) channel CaValpha2delta-1 subunit expression by the transcription factor Egr-1. Neurosci Lett.

[19] Hakki Onen S, Alloui A, Jourdan D, Eschalier A, Dubray C (2001). Effects of rapid eye movement (REM) sleep deprivation on pain sensitivity in the rat. Brain Res.

[20] Hannibal KE, Bishop MD (2014). Chronic stress, cortisol dysfunction, and pain: a psychoneuroendocrine rationale for stress management in pain rehabilitation. Phys Ther.

[21] Hao S, Mamiya K, Takahata O, Iwasaki H, Mata M, Fink DJ (2003). Nifedipine potentiates the antinociceptive effect of endomorphin-1 microinjected into the periaqueductal gray in rats. Anesth Analg.

[22] Jaaskelainen SK, Teerijoki-Oksa T, Virtanen A, Tenovuo O, Forssell H (2004). Sensory regeneration following intraoperatively verified trigeminal nerve injury. Neurology.

[23] Jeon D, Kim S, Chetana M, Jo D, Ruley HE, Lin SY, Rabah D, Kinet JP, Shin HS (2010). Observational fear learning involves affective pain system and Cav1.2 Ca2+ channels in ACC. Nat Neurosci.

[24] Korin B, Avraham S, Azulay-Debby H, Farfara D, Hakim F, Rolls A (2019) Short-term sleep deprivation in mice induces B cell migration to the brain compartment. Sleep. 10.1093/sleep/zsz222 10.1093/sleep/zsz222 PMC7616588 31553459

[25] Laumet G, Garriga J, Chen SR, Zhang Y, Li DP, Smith TM, Dong Y, Jelinek J, Cesaroni M, Issa JP, Pan HL (2015). G9a is essential for epigenetic silencing of K(+) channel genes in acute-to-chronic pain transition. Nat Neurosci.

[26] Lavand'homme P (2017). Transition from acute to chronic pain after surgery. Pain.

[27] Lee AS, Ra S, Rajadhyaksha AM, Britt JK, De Jesus-Cortes H, Gonzales KL, Lee A, Moosmang S, Hofmann F, Pieper AA, Rajadhyaksha AM (2012). Forebrain elimination of cacna1c mediates anxiety-like behavior in mice. Mol Psychiatry.

[28] Li Qi, Lu Jian, Zhou Xiaoxin, Chen Xuemei, Su Diansan, Gu Xiyao, Yu Weifeng (2019). High-Voltage-Activated Calcium Channel in the Afferent Pain Pathway: An Important Target of Pain Therapies. Neuroscience Bulletin.

[29] Li Z, Gu X, Sun L, Wu S, Liang L, Cao J, Lutz BM, Bekker A, Zhang W, Tao YX (2015). Dorsal root ganglion myeloid zinc finger protein 1 contributes to neuropathic pain after peripheral nerve trauma. Pain.

[30] Liang L, Gu X, Zhao JY, Wu S, Miao X, Xiao J, Mo K, Zhang J, Lutz BM, Bekker A, Tao YX (2016). G9a participates in nerve injury-induced Kcna2 downregulation in primary sensory neurons. Sci Rep.

[31] Liu Y, Latremoliere A, Li X, Zhang Z, Chen M, Wang X, Fang C, Zhu J, Alexandre C, Gao Z, Chen B, Ding X, Zhou JY, Zhang Y, Chen C, Wang KH, Woolf CJ, He Z (2018). Touch and tactile neuropathic pain sensitivity are set by corticospinal projections. Nature.

[32] Lu R, Fan B, Yin D, Li Y, Wang B, Zhu S, Chen Y, Xu Z (2019). Receptor for activated C kinase 1 mediates the chronic constriction injury-induced neuropathic pain in the rats’ peripheral and central nervous system. Neurosci Lett.

[33] Macrae WA (2008). Chronic post-surgical pain: 10 years on. Br J Anaesth.

[34] McAlpine CS, Kiss MG, Rattik S, He S, Vassalli A, Valet C, Anzai A, Chan CT, Mindur JE, Kahles F, Poller WC, Frodermann V, Fenn AM, Gregory AF, Halle L, Iwamoto Y, Hoyer FF, Binder CJ, Libby P, Tafti M, Scammell TE, Nahrendorf M, Swirski FK (2019). Sleep modulates haematopoiesis and protects against atherosclerosis. Nature.

[35] McCoy ES, Taylor-Blake B, Street SE, Pribisko AL, Zheng J, Zylka MJ (2013). Peptidergic CGRPalpha primary sensory neurons encode heat and itch and tonically suppress sensitivity to cold. Neuron.

[36] Mochida Sumiko (2019). Presynaptic Calcium Channels. International Journal of Molecular Sciences.

[37] Niijima F, Saito H, Murai S, Arai Y, Nakagawasai O, Tan-No K, Watanabe H, Hiraga H, Tadano T (2010). Effects of atomoxetine on levels of monoamines and related substances in discrete brain regions in mice intermittently deprived of rapid eye movement sleep. Biol Pharm Bull.

[38] Nishimoto R, Kashio M, Tominaga M (2015). Propofol-induced pain sensation involves multiple mechanisms in sensory neurons. Pflugers Arch.

[39] Onen SH, Alloui A, Gross A, Eschallier A, Dubray C (2001). The effects of total sleep deprivation, selective sleep interruption and sleep recovery on pain tolerance thresholds in healthy subjects. J Sleep Res.

[40] Pompeiano M, Cirelli C, Ronca-Testoni S, Tononi G (1997). NGFI-A expression in the rat brain after sleep deprivation. Brain Res Mol Brain Res.

[41] Qian A, Song D, Li Y, Liu X, Tang D, Yao W, Yuan Y (2013). Role of voltage gated Ca2+ channels in rat visceral hypersensitivity change induced by 2,4,6-trinitrobenzene sulfonic acid. Mol Pain.

[42] Sardi NF, Lazzarim MK, Guilhen VA, Marcilio RS, Natume PS, Watanabe TC, Lima MMS, Tobaldini G, Fischer L (2018). Chronic sleep restriction increases pain sensitivity over time in a periaqueductal gray and nucleus accumbens dependent manner. Neuropharmacology.

[43] Sardi NF, Tobaldini G, Morais RN, Fischer L (2018). Nucleus accumbens mediates the pronociceptive effect of sleep deprivation: the role of adenosine A2A and dopamine D2 receptors. Pain.

[44] Schaeffer V, Meyer L, Patte-Mensah C, Mensah-Nyagan AG (2010). Progress in dorsal root ganglion neurosteroidogenic activity: basic evidence and pathophysiological correlation. Prog Neurobiol.

[45] Tiede W, Mageri W, Baumgärtner U, Durrer B, Ehlert U, Treede RD (2010). Sleep restriction attenuates amplitudes and attentional modulation of pain-related evoked potentials, but augments pain ratings in healthy volunteers. Pain.

[46] van Loo KMJ, Rummel CK, Pitsch J, Muller JA, Bikbaev AF, Martinez-Chavez E, Blaess S, Dietrich D, Heine M, Becker AJ, Schoch S (2019). Calcium Channel subunit alpha2delta4 is regulated by early growth response 1 and facilitates Epileptogenesis. J Neurosci.

[47] Wadhwa M, Prabhakar A, Anand JP, Ray K, Prasad D, Kumar B, Panjwani U (2019). Complement activation sustains neuroinflammation and deteriorates adult neurogenesis and spatial memory impairment in rat hippocampus following sleep deprivation. Brain Behav Immun.

[48] Wang PK, Cao J, Wang H, Liang L, Zhang J, Lutz BM, Shieh KR, Bekker A, Tao YX (2015). Short-term sleep disturbance-induced stress does not affect basal pain perception, but does delay postsurgical pain recovery. J Pain.

[49] Wong CH, Wu WH, Yarmush J, Zbuzek VK (1993). An antinociceptive effect of the intraperitoneal injection of nifedipine in rats, measured by tail-flick test. Life Sci.

[50] Wright CE, Bovbjerg DH, Montgomery GH, Weltz C, Goldfarb A, Pace B, Silverstein JH (2009). Disrupted sleep the night before breast surgery is associated with increased postoperative pain. J Pain Symptom Manag.

[51] Wu CL, Raja SN (2011). Treatment of acute postoperative pain. Lancet.

[52] Xu ZZ, Kim YH, Bang S, Zhang Y, Berta T, Wang F, Oh SB, Ji RR (2015). Inhibition of mechanical allodynia in neuropathic pain by TLR5-mediated A-fiber blockade. Nat Med.

[53] Yang Y, Cui Y, Sang K, Dong Y, Ni Z, Ma S, Hu H (2018). Ketamine blocks bursting in the lateral habenula to rapidly relieve depression. Nature.

[54] Ye Y, Dang D, Viet CT, Dolan JC, Schmidt BL (2012). Analgesia targeting IB4-positive neurons in cancer-induced mechanical hypersensitivity. J Pain.

[55] Yehuda S, Sredni B, Carasso RL, Kenigsbuch-Sredni D (2009). REM sleep deprivation in rats results in inflammation and interleukin-17 elevation. J Interf Cytokine Res.

[56] Zhao X, Tang Z, Zhang H, Atianjoh FE, Zhao JY, Liang L, Wang W, Guan X, Kao SC, Tiwari V, Gao YJ, Hoffman PN, Cui H, Li M, Dong X, Tao YX (2013). A long noncoding RNA contributes to neuropathic pain by silencing Kcna2 in primary afferent neurons. Nat Neurosci.

[57] Zhuo M, Gebhart GF (2002). Modulation of noxious and non-noxious spinal mechanical transmission from the rostral medial medulla in the rat. J Neurophysiol.

