# Supplementary material for: Sleep deprivation of rats increases postsurgical expression and activity of L-type calcium channel in the dorsal root ganglion and slows recovery from postsurgical pain
Source: Acta Neuropathol Commun. 2019 Dec 23;7:217. doi: 10.1186/s40478-019-0868-2 (PMC6929318; doi:10.1186/s40478-019-0868-2)
Supplement: Supplementary file 1 — Additional file 1. Figure S1. Expression of T-type LVA channel protein did not change in the lumbar(L4-6) DRG neurons of rats subjected to perioperative SD. Figure S2. Perioperative SD increases the acitivity of HVA calcium channels and L-type calcium channels in medium/small DRG neurons at 9 days after surgery. Figure S3. Perioperative SD increases the hyperexcitability of medium/small DRG neurons at 9 days after surgery. Figure S4. Blocking L-type HVA channels in the lumbar DRG with nifedipine or a spcific shRNA does not affect response in the contralateral(control) paws. Figure S5. Specific knockdown of L-type HVA channels in the lumbar DRG inhibits total HVA-activated calcium channels and L-type calcium channels in medium/small DRG neurons at 9 days after surgery in rats subjected to perioperative SD. Table S1. Membrane input resistance and other action potential parameters in DRG neurons day 9 after incision or sham surgery. Table S2. Membrane input resistance and other action potential parameters in DRG neurons with virus injection day 9 after incision+sleep deprivation. Method S1. Viral vector mapping and sequencing report. [file 40478_2019_868_MOESM1_ESM.docx]

**Additional file 1**

**Title: Sleep deprivation of rats increases postsurgical expression and activity of L-type calcium channel in the dorsal root ganglion and slows recovery from postsurgical pain**

**Figure S1**

**
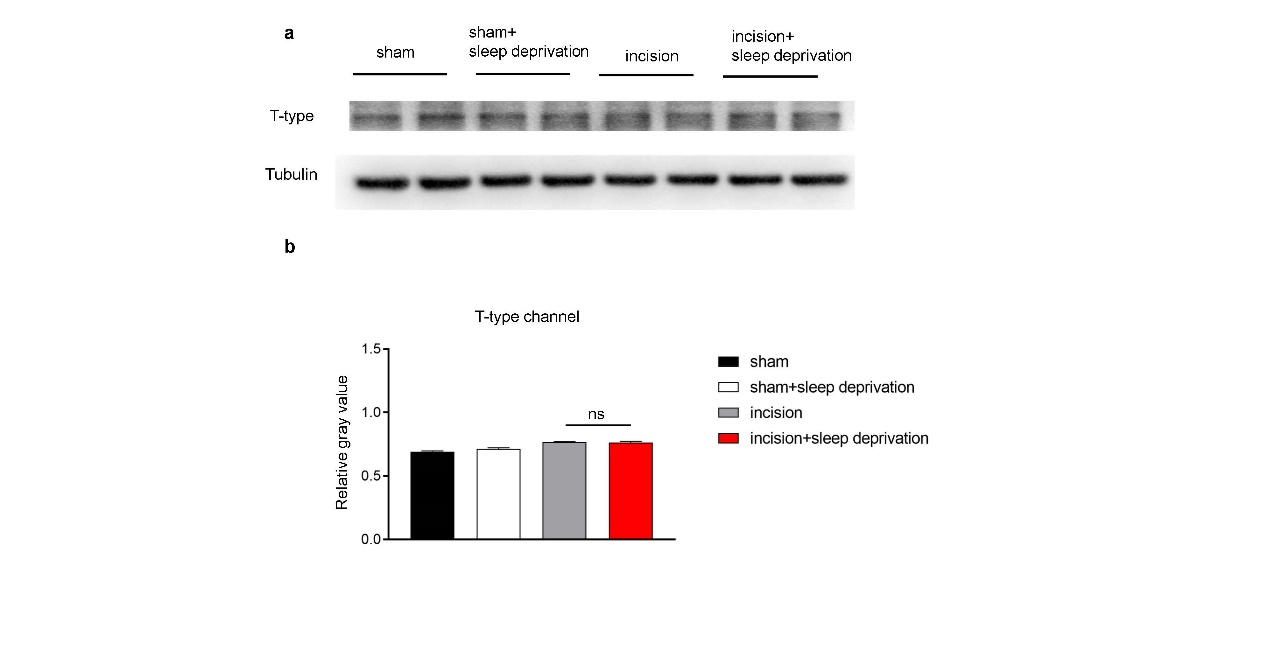
**

**Figure S1. Expression of T-type LVA channel protein did not change in the lumbar (L4-6) DRG neurons of rats subjected to perioperative SD. (a)** Western blotting of T-type channel in the L4-6 DRG of rats in four groups on day 9 after surgery. **(b)** Statistical analysis of the results in A (10 rats per group). One-way ANOVA followed by a *post hoc* Tukey test: F (3, 4) = 21.44 for the incision group *vs.* the incision+SD group.

**Figure S2**

**
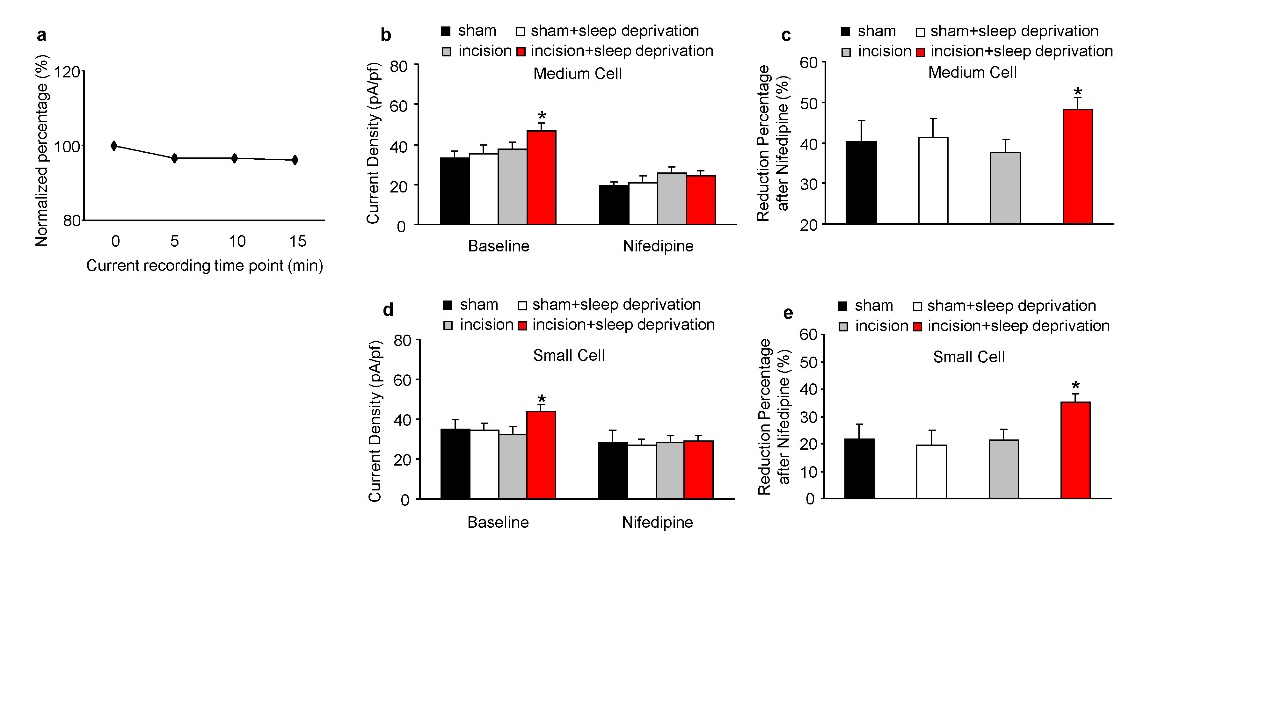
**

**Figure S2. Perioperative SD increases the activity of HVA calcium channels and L-type calcium channels in medium/small DRG neurons at 9 days after surgery. (a)** Continuous recording of total HVA calcium channels showed a minor reduction of current during the recording time. **(b)** Current density of HVA calcium channels in medium DRG neurons (5 to 6 rats per group; 11 neurons from sham group, 12 neurons from sham+SD group, 13 neurons from incision group, 16 neurons from incision+SD group). One-way ANOVA followed by a *post hoc* Tukey test: F_baseline­­_ (3, 48) = 3.282, F_nifedipine_ (3, 48) = 0.828, **p* < 0.05 for the incision group vs. the incision+SD group. **(c)** Effect of nifedipine on channel activity. Numbers of neurons and rats were the same as in B. One-way ANOVA followed by a *post hoc* Tukey test: F (3, 48) = 2.971, **p* < 0.05 for the incision group *vs.* the incision+SD group. **(d)** Current density of HVA calcium channels in small DRG neurons (5 to 6 rats per group; 11 neurons from sham group, 11 neurons from sham+SD group, 12 neurons from incision group, 13 neurons from incision+SD group). One-way ANOVA followed by a *post hoc* Tukey test: F_baseline_ (3, 43) = 3.213, F_nifedipine_ (3, 43) = 0.573, **p* < 0.05 for the incision group *vs.* the incision+SD group. **(e)** Effect of nifedipine on channel activity. Numbers of neurons and rats were the same as in D. One-way ANOVA followed by a *post hoc* Tukey test: F (3, 43) = 3.211, **p* < 0.05 for the incision group *vs.* the incision+SD group.

**Figure S3**


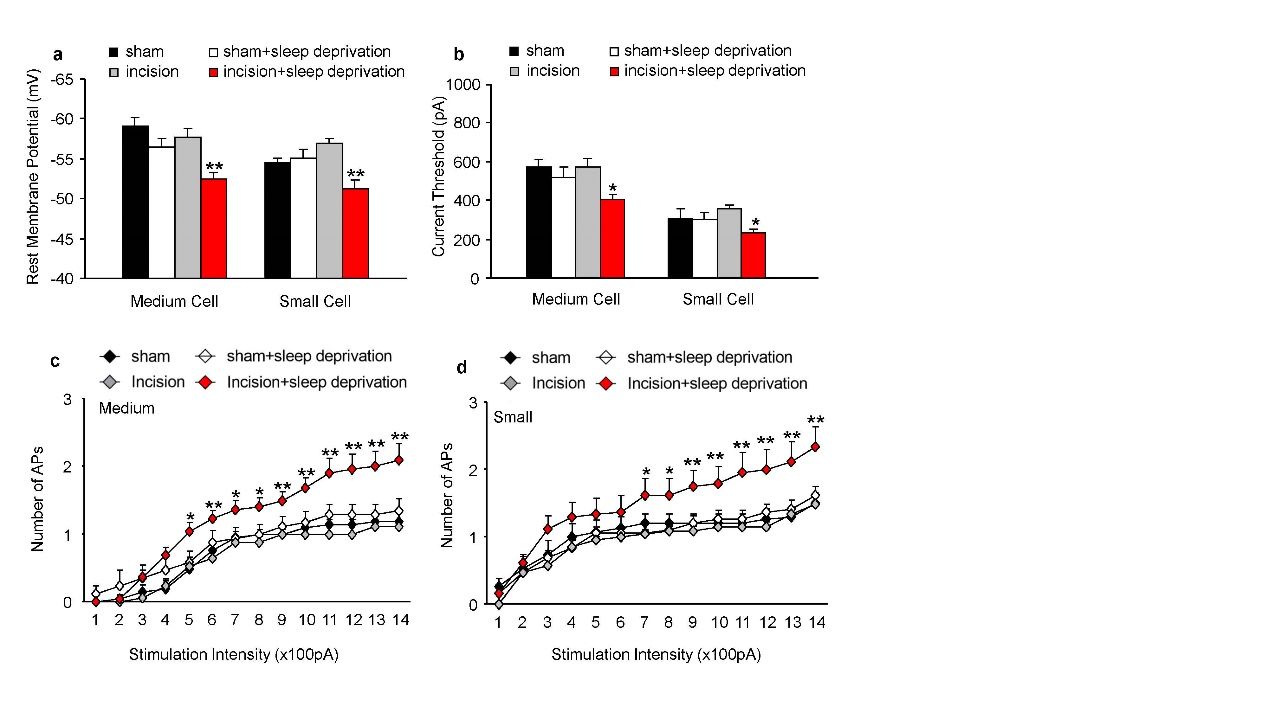


**Figure S3. Perioperative SD increases the hyperexcitability of medium/small DRG neurons at 9 days after surgery. (a)** Resting membrane potentials in medium and small DRG neurons (5 to 6 rats per group; sham group: 21 medium, 15 small neurons; sham+SD: 17 medium, 19 small neurons; incision group: 17 medium, 21 small neurons; incision+SD group: 22 medium, 24 small neurons). One-way ANOVA followed by a *post hoc* Tukey test: F (3, 73) = 9.114 for medium; F (3, 75) = 7.842 for small. ***p* < 0.01 for the incision group *vs.* the incision+SD group. **(b)** Current threshold needed to evoke the first action potential from medium, and small DRG neurons. Numbers of rats and neurons were the same as in A. One-way ANOVA followed by a *post hoc* Tukey test: F (3, 73) = 4.175 for medium; F (3, 75) = 3.323 for small. **p* < 0.05, ***p* < 0.01 for the incision group *vs.* the incision+SD group. **(c, d)** Effect of current intensity on the number of evoked action potentials in medium and small DRG neurons. Numbers of rats and neurons were the same as in A. Two-way ANOVA followed by a *post hoc* Tukey test: F_group_ (3, 1022) = 56.31 for medium (c); F_group_ (3,1050) = 41.63 for small (d). **p* < 0.05, ***p* < 0.01 for the incision group *vs.* the incision+SD group at each stimulation intensity.

**Figure S4**


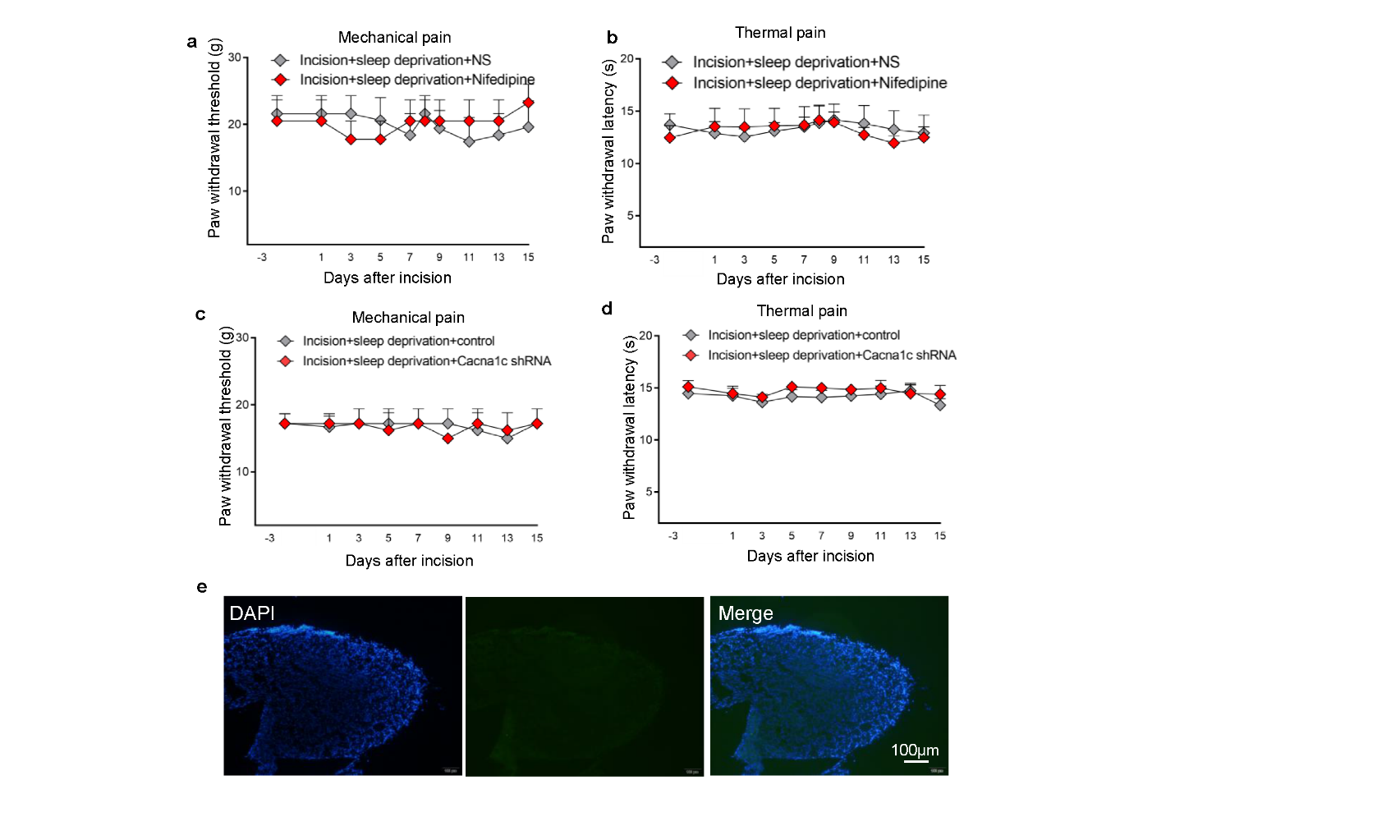


**Figure S4. Blocking L-type HVA channels in the lumbar DRG with nifedipine or a specific shRNA does not affect response in the contralateral (control) paws. (a, b)** Paw withdrawal threshold and paw withdrawal latency of contralateral (control) paws in the two groups (10 rats per group). Two-way ANOVA followed by post Sidak's multiple comparisons test: F (1, 180) = 0.021 for mechanical pain (a); F (1, 180) = 0.085 for thermal pain (b). **(c, d)** Paw withdrawal threshold and paw withdrawal latency of contralateral (control) paws (5 to 10 rats/group). Two-way ANOVA followed by post Sidak's multiple comparisons test: F (1, 95) = 0.9535 for mechanical pain (c), F (1, 72) = 3.214 for thermal pain (d). **(e)** Representative immunofluorescence slices, showing a non-injected control DRG within the same band of EGFP to omit non-specific emission. Scale bar: 100 μm.

**Figure S5**


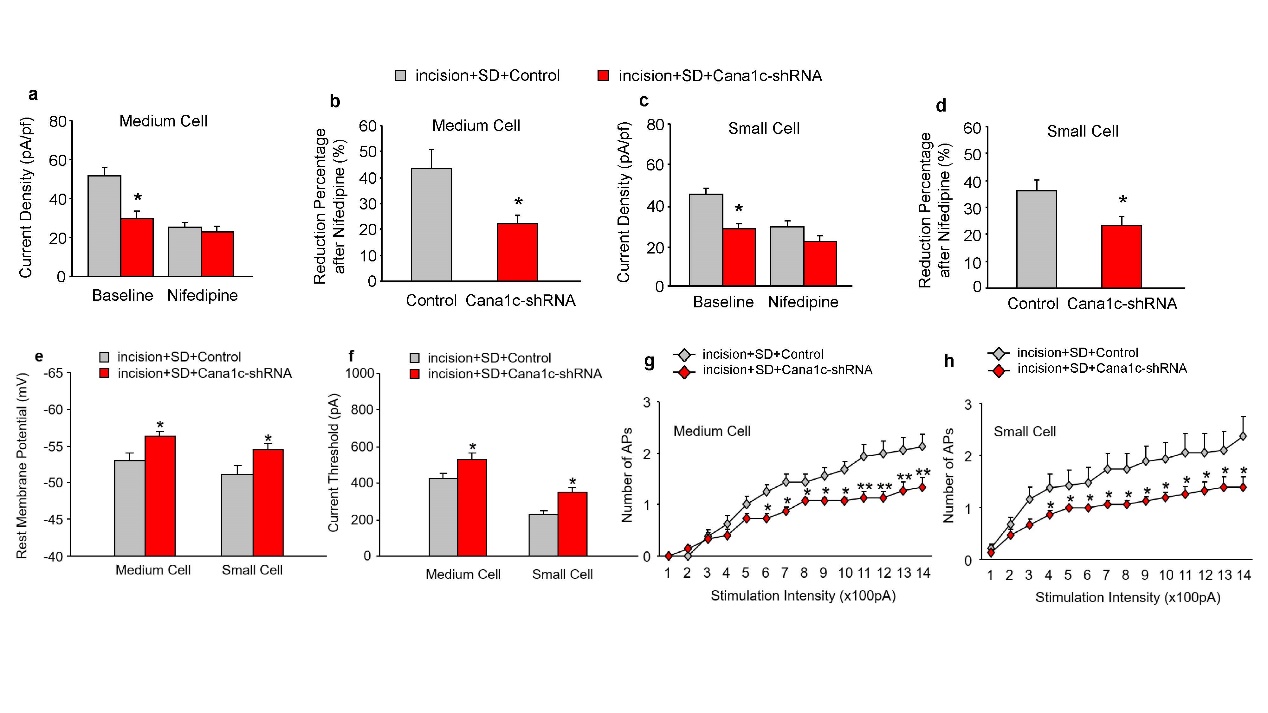


**Figure S5.** **Specific knockdown of L-type HVA channels in the lumbar DRG inhibits total HVA -activated calcium channels and L-type calcium channels in medium/small DRG neurons at 9 days after surgery in rats subjected to perioperative SD. (a)** Current density of total HVA-activated calcium channels before and after bath perfusion of 1 μm nifedipine in medium neurons (5 to 6 rats per group; n = 15 from the incision+SD +control group [grey]; n =12 medium cells from the incision+SD+shRNA group [red]). Two-tailed unpaired Student’s *t*-test: *t* = 2.347, **p* < 0.01 for comparison of the two groups. **(b)** Effect of nifedipine on percentage decrease of current density. Numbers of neurons and rats were the same as in A. Two-tailed unpaired Student’s *t*-test: *t* = 2.434, **p* < 0.05 for comparison of the two groups. **(c)** Current density of total HVA-activated calcium channels before and after bath perfusion of 1 μm nifedipine in small neurons (5 to 6 rats per group; n = 12 small cells from the incision+SD +control group [grey]; n = 12 small cells from the incision+SD+shRNA group [red]). Two-tailed unpaired Student’s *t*-test: *t* = 2.516, **p* < 0.01 for comparison of the two groups. **(d)** Effect of nifedipine on percentage decrease of current density. Numbers of neurons and rats were the same as in C. Two-tailed unpaired Student’s *t*-test: *t* = 2.434 for medium cells, *t* = 2.357 for small cells, **p* < 0.05 for comparison of the two groups. **(e)** Resting membrane potentials in medium and small DRG neurons (5 to 6 rats per group; 16 medium neurons and 19 small neurons from the incision+SD+control group; 15 medium neurons and 15 small neurons from the incision+SD+shRNA group). Two-tailed unpaired Student’s *t*-test. *t* = 2.644 for medium cells, *t* = 2.233 for small cells, **p* < 0.05 for comparison of the two groups. **(f)** Current threshold at the first action potential in medium and small DRG neurons. Numbers of neurons and rats were the same as in f. Two-tailed unpaired Student’s *t*-test: *t* = 2.464 for medium cells, *t* = 2.311 for small cells, **p* < 0.05 for comparison of the two groups. **(g, h)** Effect of simulation intensity on the number of evoked action potentials. Numbers of neurons and cells were the same as in B. Two-way ANOVA followed by a *post hoc* Tukey test: F_group_ (1, 406) = 58.87 for medium cells, F_group_ (1, 448) = 38.66 for small cells, **p* < 0.05, ***p* < 0.01 for comparison of the two groups at each stimulation intensity.

**Table S1: Membrane input resistance and other action potential parameters in DRG neurons day 9 after incision or sham surgery.**

| Large neuron | | | | | |
| --- | --- | --- | --- | --- | --- |
|  | Sham | Sham+SD | Incision | Incision+SD | F/P value |
| n | 21 cells, 6 rats | 16 cells, 5 rats | 18 cells, 5 rats | 21 cells, 6 rats |  |
| R_in_, MΩ | 46.19 ± 3.08 | 46.84 ± 9.35 | 41.30 ± 3.97 | 42.50 ± 3.61 | 0.276/0.842 |
| APT, mV | -13.54 ± 1.09 | -17.12 ± 2.12 | -12.38 ± 0.91 | -13.73 ± 1.01 | 2.202/0.095 |
| APA, mV | 98.38 ± 3.85 | 93.22 ± 2.42 | 100.10 ± 1.59 | 98.99 ± 2.51 | 1.021/0.389 |
| APO, mV | 44.84 ± 4.09 | 39.55 ± 2.88 | 42.77 ± 1.53 | 45.52 ± 2.74 | 0.710/0.549 |
| AHPA, mV | -17.38 ± 1.24 | -16.62 ± 1.13 | -14.63 ± 1.20 | -14.07 ± 0.85 | 2.090/0.109 |

Medium neuron

|  | Sham | | Sham+SD | Incision | Incision+SD | F/P value |
| --- | --- | --- | --- | --- | --- | --- |
| n | | 21 cells, 6 rats | 17 cells, 5 rats | 17 cells, 5 rats | 22 cells, 6 rats |  |
| R_in_, MΩ | | 40.80 ± 2.98 | 40.1 ± 2.92 | 46.67 ± 2.69 | 46.94 ± 2.51 | 1.732/0.168 |
| APT, mV | | -14.31 ± 1.08 | -16.35 ± 1.49 | -13.11 ± 0.76 | -13.14 ± 0.78 | 1.952/0.129 |
| APA, mV | | 100.40 ± 3.28 | 94.12 ± 3.80 | 103.8 ± 1.87 | 102.10 ± 2.21 | 1.944/0.130 |
| APO, mV | | 47.24 ± 3.32 | 41.90 ± 2.90 | 45.99 ± 1.69 | 49.57 ± 2.41 | 1.363/0.260 |
| AHPA, mV | | -17.81 ± 1.14 | -14.63 ± 1.07 | -14.93 ± 1.29 | -14.85 ± 1.26 | 1.639/0.188 |

Small neuron

|  | Sham | | Sham+SD | Incision | Incision+SD | F/P value |
| --- | --- | --- | --- | --- | --- | --- |
| n | | 15 cells, 6 rats | 19 cells, 5 rats | 21 cells, 5 rats | 24 cells, 6 rats |  |
| R_in_, MΩ | | 47.67 ± 4.79 | 46.73 ± 4.28 | 55.86 ± 3.16 | 59.27 ± 4.12 | 2.319/0.082 |
| APT, mV | | -15.42 ± 1.48 | -19.16 ± 2.08 | -15.15 ± 0.83 | -15.15 ± 1.07 | 1.956/0.128 |
| APA, mV | | 92.48 ± 1.78 | 91.06 ± 3.09 | 96.31 ± 1.15 | 97.97 ± 2.09 | 2.305/0.084 |
| APO, mV | | 38.96 ± 2.33 | 39.40 ± 3.81 | 39.34 ± 1.38 | 46.73 ± 1.84 | 2.663/0.054 |
| AHPA, mV | | -13.24 ± 1.12 | -14.02 ± 1.43 | -16.86 ± 0.98 | -16.12 ± 1.01 | 2.103/0.107 |

Values are Mean ± S.E.M., SD: Sleep Deprivation. R_in_: membrane input resistance. APT: action potential threshold. APA: action potential amplitude. APO: action potential overshoot. AHPA: afterhyperpolarization amplitude. All values are mean±S.E.M. One way ANOVA test.

**Table S2: Membrane input resistance and other action potential parameters in DRG neurons with virus injection day 9 after incision + sleep deprivation.**

|  | Large neurons | | | Medium neurons | | | Small neurons | | |
| --- | --- | --- | --- | --- | --- | --- | --- | --- | --- |
|  | Control | shRNA | t/p value | Control | shRNA | t/p value | Control | shRNA | t/p value |
| n | 18 cells, 6 rats | 13 cells, 5 rats |  | 16 cells, 5 rats | 15 cells, 6 rats |  | 19 cells, 5 rats | 15 cells, 6 rats |  |
| R_in_, MΩ | 44.55 ± 3.89 | 47.61 ± 5.23 | 0.481/0.634 | 48.78 ± 2.94 | 49.15 ± 5.66 | 0.059/0.953 | 61.17 ± 4.73 | 52.39 ± 5.31 | 1.234/0.226 |
| APT, mV | -13.91 ± 1.16 | -15.76 ± 0.88 | 1.180/0.248 | -13.29 ± 0.79 | -16.03 ± 1.49 | 1.658/0.108 | -15.55 ± 1.29 | -18.56 ± 1.93 | 1.341/0.189 |
| APA, mV | 99.32 ± 2.31 | 92.17 ± 3.70 | 1.173/0.095 | 101.60 ± 2.68 | 94.17 ± 2.76 | 1.926/0.064 | 97.30 ± 2.53 | 90.09 ± 3.19 | 1.795/0.082 |
| APO, mV | 45.50 ± 2.73 | 37.63 ± 3.26 | 1.186/0.074 | 48.55 ± 2.93 | 41.83 ± 2.40 | 1.761/0.089 | 46.20 ± 2.22 | 40.22 ± 2.76 | 1.710/0.097 |
| AHPA, mV | -13.82 ± 0.97 | -12.25 ± 0.81 | 1.181/0.247 | -14.11 ± 1.58 | -13.67 ± 1.30 | 0.215/0.832 | -16.22 ± 1.19 | -13.91 ± 0.93 | 1.460/0.154 |

Values are Mean ± S.E.M., SD: Sleep Deprivation. R_in_: membrane input resistance. APT: action potential threshold. APA: action potential amplitude. APO: action potential overshoot. AHPA: afterhyperpolarization amplitude. All values are mean±S.E.M. Two-tailed unpaired students’ t-test.

**Supplemental Method**

**Viral vector mapping and sequencing report**

1. vector information

element order: pAAV2-H1-shRNA-CAG-eGFP

clone site: Esp3I


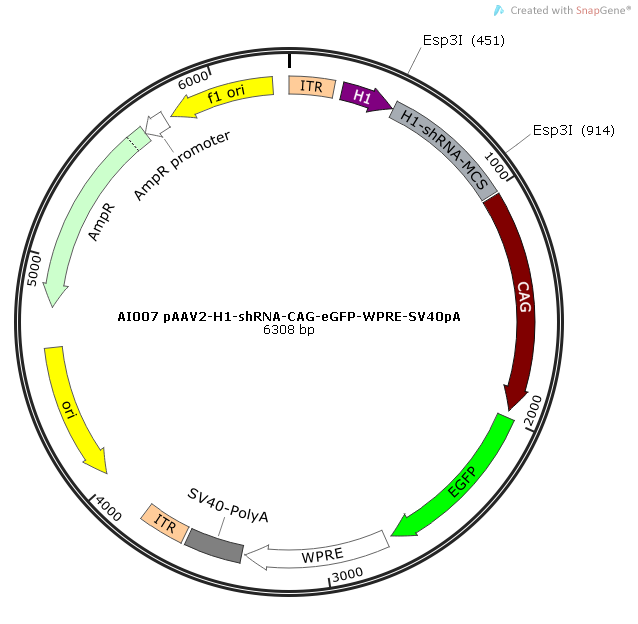


1. Enzyme digestion identification of recombinant plasmid

enzyme cutting site: SmaI

expected strip size: 3309 bp + 2575 bp + 11 bp（off the gel） + 11 bp（off the gel）

1 2 3


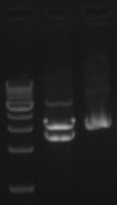


#1: 1kb DNA Marker, stripe from down to up: 1kb, 2kb, 3kb, 4kb, 5kb, 6kb, 7kb, 8kb, 9kb, 10kb

#2: After the recombinant plasmids were digested with SmaI enzyme, the strips basically met the expectation

#3: Control of the recombinant plasmids without enzyme digestion

Enzyme digestion identification results: the size of the strips were in line with the expectation

1. The recombinant plasmid sequencing

The recombinant plasmids verified by enzyme digestion were sent for sequencing. The effective length of sequencing is about 800 bp. The effective length is affected in many ways, so the validity and accuracy of the plasmids were determined based on the peak map. After the comparision, the primer sequence can be found on the sequencing result, indicating that the sequence insertion is successful.
